# Is large-scale rapid CoV-2 testing a substitute for lockdowns?

**DOI:** 10.1371/journal.pone.0265207

**Published:** 2022-03-18

**Authors:** Marc Diederichs, René Glawion, Peter G. Kremsner, Timo Mitze, Gernot J. Müller, Dominik Papies, Felix Schulz, Klaus Wälde

**Affiliations:** 1 Johannes Gutenberg University Mainz, Mainz, Germany; 2 University of Hamburg, Hamburg, Germany; 3 University of Tübingen, Tübingen, Germany; 4 Centre de Recherches Médicales de Lambaréné, Lambaréné, Gabon; 5 University of Southern Denmark, Odense, Denmark; 6 CESifo, Munich, Germany; 7 CEPR, London, United Kingdom; 8 IZA, Bonn, Germany; Xiamen University, CHINA

## Abstract

**Background:**

Various forms of contact restrictions have been adopted in response to the Covid-19 pandemic. Around February 2021, rapid testing appeared as a new policy instrument. Some claim it may serve as a substitute for contact restrictions. We study the strength of this argument by evaluating the effects of a unique policy experiment: In March and April 2021, the city of Tübingen set up a testing scheme while relaxing contact restrictions.

**Methods:**

We compare case rates in Tübingen county to an appropriately identified control unit. We employ the synthetic control method. We base interpretations of our findings on an extended SEIR model.

**Findings:**

The experiment led to an increase in the reported case rate. This increase is robust across alternative statistical specifications. This is also due to more testing leading initially to more reported cases. An epidemiological model that corrects for ‘more cases due to more testing’ and ‘reduced testing and reporting during the Easter holiday’ confirms that the overall effect of the experiment led to more infections.

**Interpretation:**

The number of rapid tests were not sufficiently high in this experiment to compensate for more contacts and thereby infections caused by relaxing contact restrictions.

## Introduction

Can large-scale CoV-2 testing strategies substitute for restrictive public health measures? In theory, the idea is straightforward. If, first, every socially active person is subjected to a rapid CoV-2 test on a regular basis and, second, quarantined if tested positive, there is (almost) zero infection risk from social interactions. One would achieve the same outcome as under a complete lockdown—albeit at much lower costs: social interactions could be maintained.

In practice, there are several complications. Any testing procedure generates false negatives, that is, some infections will necessarily go undetected [1]. Moreover, the timing of testing is critical: when testing takes place too early, infected persons go undetected, when it takes place too late, the transmission of the disease may have already taken place. Some therefore suggest that rapid tests do more harm than good [2]. Lastly, testing and quarantining may be not sufficiently comprehensive, for instance, because of a lack of compliance.

Lockdowns, on the other hand, are also unlikely to prevent new infections altogether. First and foremost, they cannot be complete because some social interactions are essential. Second, their effectiveness also suffers from lack of compliance [3, 4].

Given this debate, an empirical assessment seems warranted. This paper turns to a uniquely suited policy experiment set up in the German town of Tübingen. Between March 16 and April 24, 2021, it ran a large-scale rapid testing scheme while simultaneously relaxing lockdown measures (Section A1 in S1 Appendix). Each negatively-tested person was permitted, inter alia, to shop, go to movie theaters or join other people in restaurants (outdoors). While other towns tried to obtain similar permits elsewhere in Germany [5], the case of Tübingen is unique as its experiment started while other German counties were still in lockdown. We rely on these counties as a reference group in our synthetic control method [6–9] to assess whether large-scale rapid CoV-2 testing can be a substitute for lockdowns. The answer would be yes if opening under safety did not increase cases in Tübingen.

## Results

### Empirical findings

**Fig 1:**
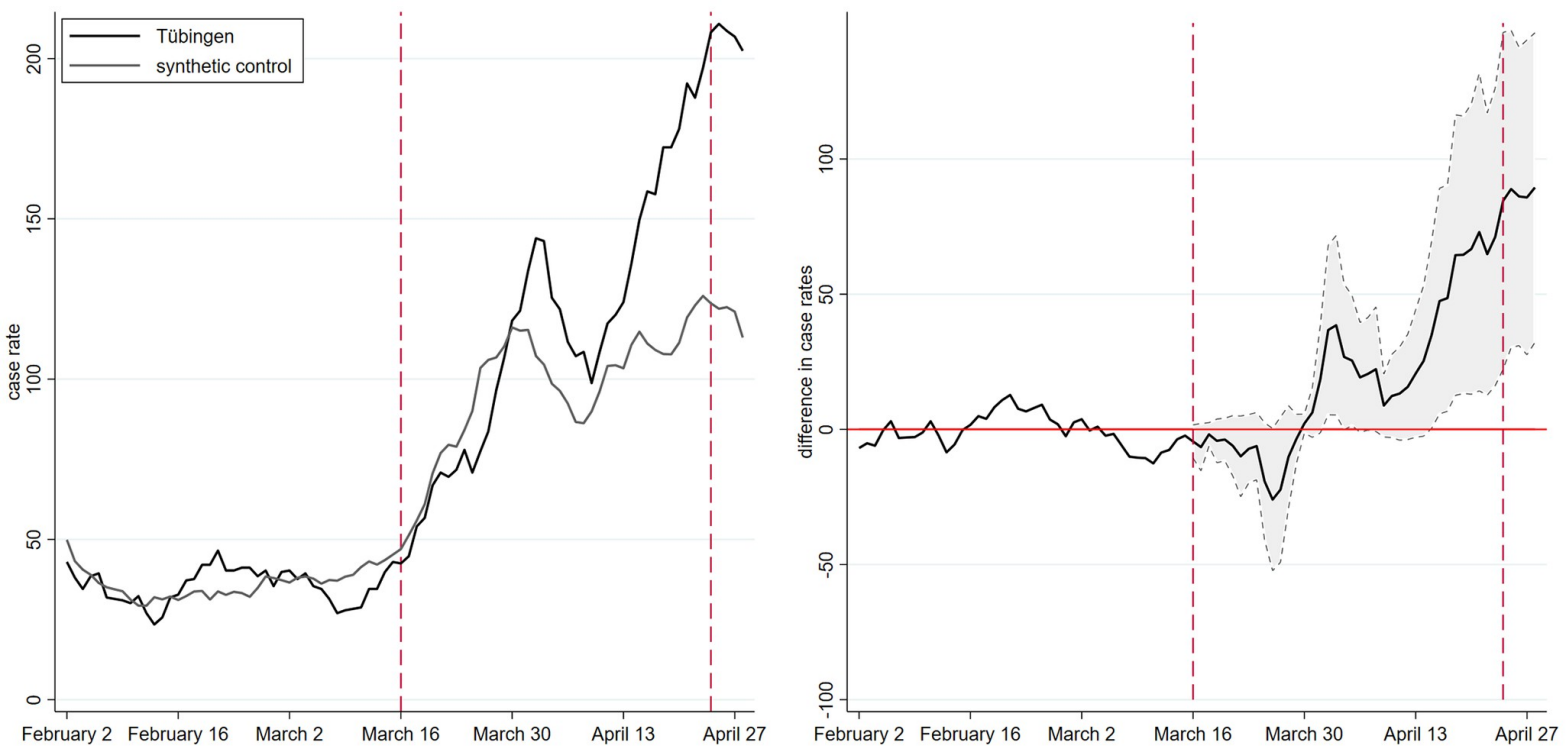
Seven-day case rates of Tübingen and control group.

We describe the pandemic state by the key metric for policy decisions in Germany: the seven-day SARS-CoV-2 case rate (Section A2.1 in S1 Appendix). The left panel in Fig 1 shows the development of the case rate between February and April 2021. The solid black line in the left panel represents the development in Tübingen county, the dashed red vertical lines indicate the start and end of the policy experiment. The line for Tübingen county shows that the case rate was below 50 before the start of the project and increased to almost 150 during the Easter weekend starting April 2. This increase coincided with opening under safety (OuS) and led to wide public claims that “Tübingen failed”.

Statistics tells us, however, that we cannot assess the causal effect of a policy experiment by comparing the case rate before and after the start of the project. Other factors than OuS are likely to have affected pandemic dynamics in Tübingen over this period as well. We, therefore, need to compare the pandemic development in Tübingen county to a control group of similar counties: counties should display comparable pandemic dynamics before the start of OuS in Tübingen, should share certain fundamental socio-demographic and health care characteristics (e.g., population density, age structure, medical services, commuting patterns) and should be subject to very similar if not identical public health measures.

We identify such a set of control counties using the synthetic control method (see section Methods). The resulting control counties and their weights constituting our synthetic control county are presented in Table 1. The synthetic control county consists of four urban districts (‘Stadtkreis, SK’) and four rural districts (‘Landkreis, LK’). Two of the three units that receive the largest weights (Freiburg and Heidelberg) are cities in Baden-Württemberg with major universities that have similar population levels of up to 230K and comparable socio-demographic structures. Local health care systems are also similar. Table A4.4 in S1 Appendix shows the details of the fit.

**Table 1:** Control counties and their weights for Fig 1.

name	weight	name	weight
SK Freiburg i.Breisgau	0.29	LK Pfaffenhofen a.d.Ilm	0.07
LK Eichstätt	0.24	LK Bitburg-Prüm	0.05
SK Heidelberg	0.19	SK Münster	0.04
SK Oldenburg	0.09	LK Lüneburg	0.03

Given this background, we can now again turn to Fig 1. The solid black line, representing Tübingen county, and the grey line, representing the synthetic control, show very similar case rates prior to the beginning of the experiment, indicating a good fit in the pre-treatment period since February 2021. When we compare the development in reported case rates after the beginning of the experiment, we initially observe a parallel development between Tübingen county and its synthetic twin. At the beginning of April, cases in the control county start to decline, whereas the decline in Tübingen county only sets in a few days later. By April 10, the gap between Tübingen county and the control county is almost closed.

This development is also visible in the right panel of Fig 1. A first peak in the difference between treatment and control occurs 2.5 weeks after the start around April 3, just around the Easter weekend. The right panel also shows that this difference, while visible, is hardly statistically different from zero at the 10% level. Nevertheless, a treatment effect is visible: OuS seems to increase the case rate—at least temporarily.

As of April 10, however, data not included in our earlier version [10], case rates in Tübingen more strongly increase relative to its synthetic twin. While some open questions related to OuS in Tübingen will be addressed in our discussion section, the most straightforward interpretation of this increase after April 10 is the continuation of a process OuS initiated on March 16: More contacts lead to more cases. The reduction in the gap between Tübingen and its synthetic twin is due to the Easter holidays and the slowdown of reporting of data from laboratories and doctors to local and national health authorities.

### Testing and the Easter break

Any empirical finding calls for a theoretical interpretation. Empirically, we find that OuS increases case rates. Theoretically, at least two questions arise: Did case rates increase only because OuS implies more testing and when we test more, we find more? Second, can we believe our verbal interpretation that OuS and the Easter break imply such a non-monotonic behavior as visible in Fig 1? We answer these questions in turn.

From a behavioural perspective, a third issue arises. One might conjecture that individuals take too many risks (i.e. allow for too many social contacts) as they feel “too secure” due to a negative test. We discuss this insurance issue related to the Peltzman-effect [11] in section ‘Were there too many social contacts?’.

#### Case rates and testing

Let us first turn to the question whether case rates increase only because OuS implies more testing. Some do indeed argue that the number of reported infections increases when there is more testing. The argument is not convincing when a test is undertaken because a patient with Covid-19 symptoms visits a doctor. If doctors arrange for tests, the number of tests depends on the number of patients with Covid-19 symptoms. The number of reported infections therefore increases only when there are more patients with symptoms. Tests increase as a function of the state of the pandemic [12].

The argument is true when testing is the outcome of projects such as OuS. In this case, the number of tests does not depend on the state of the pandemic but on the number of participants that want to be tested. Similar arguments can be made with respect to testing travelers, testing sport professionals, or all other preventive testings. In this case, more infected individuals are found when there is more testing.

To understand the quantitative importance of this argument for OuS, we extend a standard SIR model in four respects (Section A5.2 in S1 Appendix). We (a) allow for rapid testing leading to (b) discovery of asymptomatic (unreported) cases who, thereby, (c) turn into reported cases. We assume that (d) reported infectious individuals are in quarantine and infections can only occur when meeting a non-reported infectious individual [13]. OuS in this framework consists of these four features plus an increase in the contact rate, i.e., as an example, the number of individuals one person meets per day.

When we want to separate the effect of more contacts from the effect of more testing, we first fit the extended SIR model to the data (Section A5.2 in S1 Appendix). Second, we switch off the testing channel by assuming that no extra testing takes place: in this case, the model predicts that, all else equal, the increase in (reported) cases is less strong initially (Section A4.2 in S1 Appendix). This finding supports the notion that more testing leads to more (reported) cases. However, the effect is quantitatively small (with a maximum of 7%) and vanishes over time because the increase in case rates accelerates in the absence of testing (and quarantining). Hence, the fact that more testing leads to more cases is unlikely to be the reason for the strong increase in the case rate in Tübingen (Fig 1) in the context of OuS.

#### OuS and Easter break

We now turn to our verbal interpretation that OuS and the Easter break imply a non-monotonic behavior as visible in Fig 1. Our complete explanation of Fig 1 builds on a combination of a (i) permanent OuS effect and a (ii) temporary Easter break effect. OuS has a permanent effect on the contact rate, the Easter break temporarily reduces reporting and testing. We capture these two effects by an additional feature of our SIR model (Section A5.2 in S1 Appendix) that lets the flow from exposed to reported infectious individuals fall over Easter. A certain share of infectious individuals who display symptoms do not go to an emergency center and therefore do not get tested.

Employing this framework shows that one can easily explain the rise in case rates in Tübingen by an increase in contact rates (Section A4.3 in S1 Appendix). The temporary drop over the Easter break can be understood by a reduction in transmission and testing. This finding holds when we estimate one unique increase of contacts and when we estimate two separate contact rates, one before and one as of Easter (Table A4.5 in S1 Appendix). We concede that the Easter effect in the SIR model is not as pronounced as in the data. What we can clearly see, however, is that the permanent effect of OuS can easily explain the entire increase in case rates. Our theoretical interpretation therefore confirms our empirical findings. OuS did increase case rates in Tübingen relative to its control group.

#### Were there too many social contacts?

The increase of reported cases is likely to be caused by increased social interactions. Under OuS, there are (at least) two reasons for why social interactions increase with testing. First, OuS mandates testing as a prerequisite for social interactions such as shopping. Second, people may in addition intensify their social interactions if they and their peers are tested more frequently because more frequent testing reduces the perceived risk of becoming infected. In economics such a behavioral adjustment is known as “risk compensation”, following an influential study by Peltzman [11]. Peltzman hypothesized that because of a given “demand for safety”, people adjust their driving behavior in response to legally mandated safety devices such as seat belts in automobiles to the extent that their driving behavior becomes riskier. Whether the behavioral adjustment completely offsets the direct effect of the regulation has been debated ever since and in various contexts, including the transmission of diseases [14–16]. There appears to be a consensus that the behavioral adjustment can somewhat reduce the effects of regulations without offsetting them entirely. Based on our assessment of the literature (see also, e.g., [17, 18]), we conclude that the behavioral adjustments go some way towards accounting for our findings and thus may contribute to the effects that we identify in this study.

## Methods

### Implementing the synthetic control method

We estimate the causal effect of OuS (the ‘treatment’) on infection dynamics in Tübingen (the ‘treated unit’) by relying on the synthetic control method (SCM). It was proposed for the causal assessment of policy interventions based on aggregate outcome measures [6, 7]. At the heart of this method lies an estimator which identifies, in our application, counties in Germany to which Tübingen county can be compared (the ‘synthetic control county’). This comparison is based on information observable prior to treatment and summarized by a set of predictor variables (the ‘predictor set’). SCM requires an a-priori list of counties (the ‘donor pool’) from which to construct the control unit. See Section A5.1 and Table A4.4 in S1 Appendix for more background.

Results depend on how we measure the pandemic (the ‘outcome variable’). Our preferred outcome variable is the 7-day case rate. We also employ cumulative cases and, briefly, the positive rate, as alternatives. Robustness checks for the predictor set, the donor pool and outcome variables are undertaken and will be discussed shortly.

The present study puts special emphasis on two novel predictor variables. First, we allow for spatial controls. They are due to the low case rate of Tübingen compared to other counties in Germany before the start of OuS on March 16, visible in Fig A2.2 in S1 Appendix. If visitors enter Tübingen from counties with higher case rates, Tübingen will likely experience higher case rates itself. To this end, we looked for control counties that were also surrounded by counties with case rates similar to the neighbors of Tübingen. If Tübingen is subject to a ‘catching up’ process, we wanted to make sure that Tübingen is compared with regions that are also subject to ‘catching up’.

Second, it appears very important that counties are as similar as possible to Tübingen county in terms of Covid-19 policies. We achieved this goal in two ways. On the one hand, we constructed an index (see Section A2.4 in S1 Appendix) that measures the stringency of Covid-19 policies. As an alternative, we compare Tübingen to counties from its state Baden-Württemberg only. Counties all coming from the same state as Tübingen are very homogeneous with respect to their Covid-19 rules.

We also inquired into the robustness of our findings by re-estimating the effects employing differences-in-differences. The results are basically identical to our SCM findings. See section A9.2 of the S1 Appendix for a detailed discussion of the method and the findings.

### Estimating an extended SIR model

The differential effects of ‘opening’ and of ‘safety’ (i.e. testing) in OuS can be understood and quantified by estimating an extended SIR model [19–21]. Our central extension of a standard SIR model (Section A5.2 in S1 Appendix) consists in modeling rapid testing and the Easter break.

Testing implies a flow from asymptomatic, non-reported individuals to reported individuals. Assuming that all reported individuals enter quarantine, the share of infectious individuals in society (one can meet) falls due to testing. The Easter break implies that (i) test results are not reported to health authorities and that (ii) not all individuals with symptoms visit doctors.

We quantify the parameters of both extensions by matching cumulative cases, case rates and observed positive tests. We do so by minimizing the squared difference between data and model predictions [20]. We infer the effects of testing on the reported number of infections by computing a hypothetical time series for cases under the assumption that no testing was undertaken in Tübingen. The effect of the Easter break is quantified by estimating the share of individuals that do not visit emergency units despite having symptoms.

## Discussion

Findings from a comparison of a county with a synthetic county depend on (a) the measure used (outcome variable), (b) the criteria employed to find comparable counties (predictor set) and (c) the group of counties from which to choose comparable counties (donor pool). Varying our choices confirms the basic finding.

### Predictor sets and donor pool

**Fig 2:**
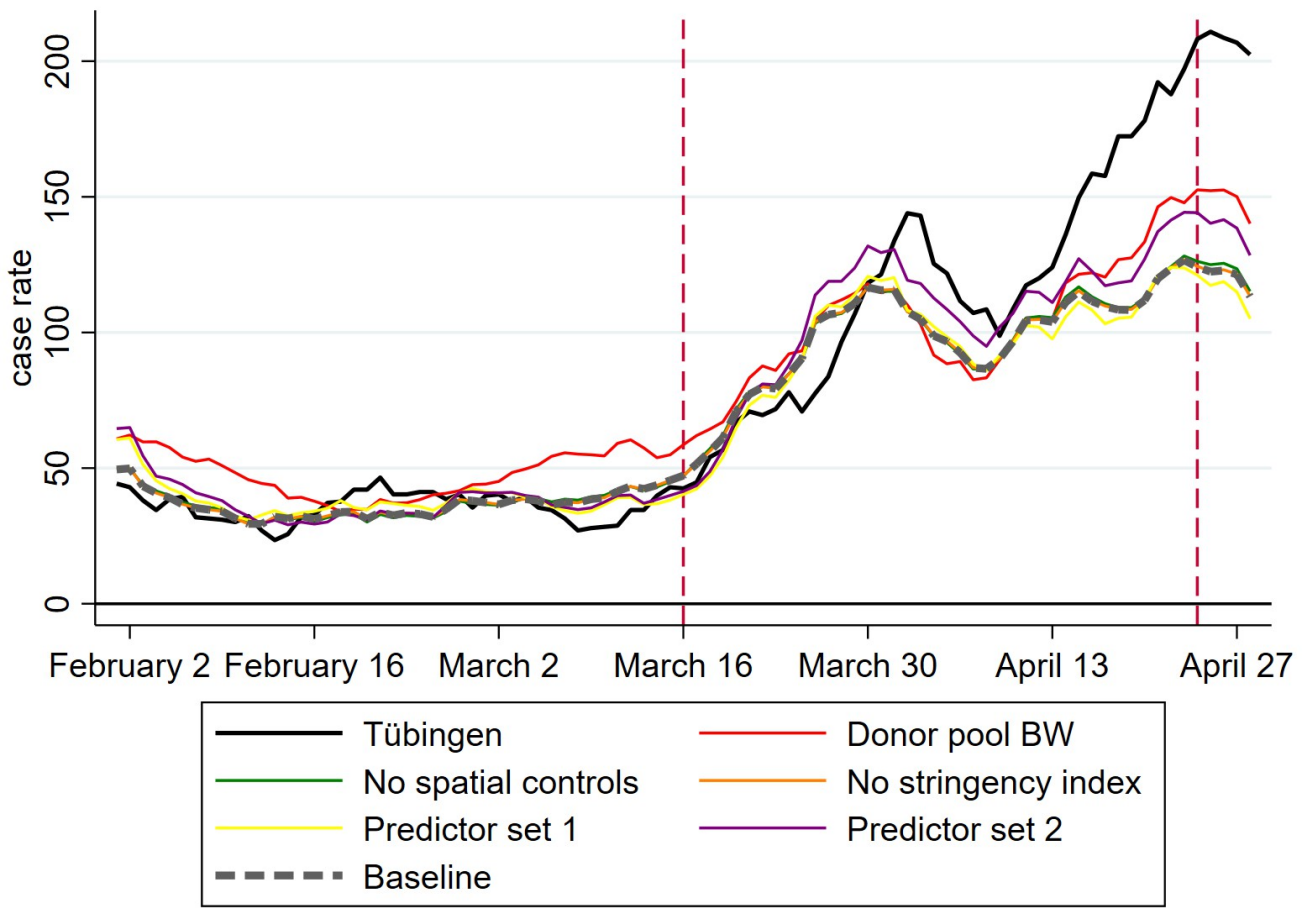
Seven-day case rates for alternative predictor sets and donor pools.

The outcome of our main robustness checks for Tübingen county are visible in Fig 2. In addition to our predictor set ‘baseline’, employed for estimating results displayed in Fig 1, we structure our discussion around two additional predictor sets, a predictor set 1 and a predictor set 2. Our baseline predictor set, visible in Table A4.4 in S1 Appendix, was discussed in the method section. Predictor sets 1 and 2 (visible in Tables A8.13 and A8.15 in S1 Appendix, respectively) shorten the pre-treatment period, starting on February 1 in the baseline predictor set, to a start date of March 1. This investigates into the importance of pandemic pre-treatment variables for the pre-treatment fit and for the overall result. Predictor set 1 employs daily case rates while predictor set 2 employs weekly case rates. Predictor set 2 therefore further reduces the importance of pre-treatment pandemic measures relative to non-pandemic variables. To understand the importance of spatial controls and of the stringency index, we employ a predictor set defined as the baseline predictor set without the stringency index and another one defined as baseline without spatial controls.

As Fig 2 shows, all of our robustness analyses confirm the baseline scenario. This is most impressively visible by the hardly visible (grey dashed) baseline graph in this figure: the predictor set without the stringency index leads to basically the same result. When we take out spatial controls, the prediction gets slightly better. This means that the ‘catching up’ argument laid out in the method section does not have a large quantitative importance. The shorter pre-treatment fitting period of predictor set 1 leads to a somewhat worse prediction than baseline. Again, quantitatively, this is of no importance.

Going from daily to weekly pre-treatment frequency with predictor set 2 worsens the pre-treatment fit but improves the post-treatment prediction. The same is true when we restrict the donor pool to Baden-Württemberg only. If we therefore put more emphasis on homogeneity in Covid-19 policy, we could tell a somewhat more optimistic story. Given the worse pre-treatment fit (RMSPE of 19.57 in Table A6.9 in S1 Appendix instead of 9.9 in Table A4.4 in S1 Appendix), however, we do not put too much emphasis on this finding. We conclude that our baseline result is confirmed by these robustness tests that vary the predictor set and the donor pool.

### The role of the pandemic measure

The seven-day case rate as employed in Fig 1 is the measure of the pandemic state that receives most of the attention around the world. It is not clear, however, whether this is the best measure for a pandemic. It is also not clear whether this is the best measure to compare the evolution of the pandemic across regions. A moving average over a period of seven days is much more short-run in nature than, for example, the sum of all new infections since some starting point.

We therefore employ the total number of reported infections since January 2021 per 100,000 inhabitants as dependent variable. Section A7 in S1 Appendix shows that the synthetic twin of Tübingen consists of different counties than in our benchmark analysis. The fit dominates the baseline fit as cumulative infections over a longer period than only seven days are less volatile. Finding similar counties is therefore easier for the SCM. What is most important, however, is the evaluation of OuS: We confirm the findings from above. Interestingly, Tübingen and its control also move more or less in parallel up to around April 1. Only then, the difference becomes much larger, just as in the case of the case rates.

An alternative popular measure of the severity of a pandemic is the positive rate, i.e. the share of positive tests in the total number of tests being undertaken in a certain population. This was the official measure of the scientific team behind OuS in Tübingen (of which one of us was the head) and of policy-makers. By this measure, there was no increase in the severity of the pandemic in Tübingen. Local outbreaks in communities and residential homes might have increased the case rate in the county but were not visible in the positive rate.

### Is Tübingen city different?

Many commentators on OuS in Tübingen have argued that conclusions drawn from Tübingen county could be misleading. One should rather study Tübingen city. Probably findings on OuS would be less negative in this case, the claim goes.

The details of the timing of OuS in Tübingen are in Table A1.1 in S1 Appendix. All testing and opening measures took place in the city of Tübingen. As of April 6, only shopping in non-essential stores for local residents continued. Those parts of the experiments that attracted most visitors had ended. Maybe the increase in case rates in Tübingen county is due to increases in communities other than Tübingen city (see Fig A10.17 in S1 Appendix for a map).

To study this hypothesis, we first look at the evolution of case rates in Tübingen city, also shown in Fig A2.2 in S1 Appendix in addition to Tübingen county. We observe that Tübingen city had indeed an (even) lower level of case rates than Tübingen county. We also see that Tübingen city followed the same overall trend as Tübingen county. What is more, Fig A2.3 in S1 Appendix shows that *increase* in case rates in Tübingen city is larger than the increase in Tübingen county. This raises some doubts about the hypothesis.

To be on the safe side, we treat Tübingen city as an independent county and apply the SCM again. We adjust the predictor set (as different data are available at the community level than at the county level), the donor pool and the pandemic measure (see Section A10 in S1 Appendix). We also adjust the pandemic measure as Tübingen city had one of the lowest, if not the lowest, case rates in all of Germany before the treatment date. This makes it impossible to construct an appropriate synthetic twin employing the case rate as pandemic measure. The new pandemic measure we use for Tübingen city is therefore the growth factor for case rates.

Before proceeding, we confirm that the use of a new predictor set and a new pandemic measure do not strongly affect our findings: We compare our baseline findings with findings based on the new predictor set and the new pandemic measure. Applying SCM then finally to Tübingen city shows that Tübingen city experienced a stronger increase in (growth of) case rates than its control regions.

Returning to the hypothesis, it is true that the increase in case rates in other communities belonging to Tübingen county contributed to the rise of cases in Tübingen county. There is just as much truth to the finding, however, that Tübingen city experienced a rise in case rates as well and that this rise is larger than the increase in comparable control counties.

## Supporting information

S1 Appendix Appendix: Is large-scale rapid CoV-2 testing a substitute for lockdowns?. (PDF)

## References

[1] Dinnes J, Deeks JJ, Berhane S, Taylor M, Adriano A, Davenport C, et al. Rapid, point-of-care antigen and molecular-based tests for diagnosis of SARS-CoV-2 infection. Cochrane Database of Systematic Reviews. 2021;(3). doi: 10.1002/14651858.CD013705.pub2 33760236 PMC8078597

[2] Guglielmi G. Rapid coronavirus tests: a guide for the perplexed; 2021. 10.1038/d41586-021-00332-4 33564189

[3] Graffigna G, Barello S, Savarese M, Palamenghi L, Castellini G, Bonanomi A, et al. Measuring Italian citizens’ engagement in the first wave of the COVID-19 pandemic containment measures: A cross-sectional study. PLOS ONE. 2020;15(9). doi: 10.1371/journal.pone.0238613 32915822 PMC7485890

[4] Masters NB, Shih SF, Bukoff A, Akel KB, Kobayashi LC, Miller AL, et al. Social distancing in response to the novel coronavirus (COVID-19) in the United States. PLOS ONE 15(9): e0239025. 2020;. doi: 10.1371/journal.pone.0239025 32915884 PMC7485770

[5] Diederichs M, Mitze T, Schulz F, Wälde K. Testing & Opening in Augustusburg—A Success Story? https://wwwmacroeconomicsuni-mainzde/klaus-waelde/ongoing-work-and-publications/. 2021;.

[6] Abadie A, Diamond A, Hainmueller J. SYNTH: Stata module to implement Synthetic Control Methods for Comparative Case Studies; 2011. Statistical Software Components, Boston College Department of Economics. Available from: https://ideas.repec.org/c/boc/bocode/s457334.html.

[7] Abadie A, Diamond A, Hainmueller J. Comparative Politics and the Synthetic Control Method. American Journal of Political Science. 2015;59(2):495–510. doi: 10.1111/ajps.12116

[8] Mitze T, Kosfeld R, Rode J, Wälde K. Face masks considerably reduce COVID-19 cases in Germany. Proceedings of the National Academy of Sciences. 2020;117(51):32293–32301. doi: 10.1073/pnas.2015954117 33273115 PMC7768737

[9] Born B, Dietrich AM, Müller GJ. The lockdown effect: A counterfactual for Sweden. PLOS ONE. 2021;16(4):1–13. doi: 10.1371/journal.pone.0249732 33831093 PMC8031244

[10] Diederichs M, Kremsner PG, Mitze T, Müller GJ, Papies D, Schulz F, et al. Is large-scale rapid CoV-2 testing a substitute for lockdowns? The case of Tübingen. medRxiv. 2021; 10.1371/journal.pone.0265207 PMC8932588 35302989

[11] Peltzman S. The Effects of Automobile Safety Regulation. Journal of Political Economy. 1975;83(4):677–725. doi: 10.1086/260352

[12] Wälde K. How to remove the testing bias in CoV-2 statistics. IZA DP. 2020;No. 13785, http://ftp.iza.org/dp13785.pdf.

[13] Jones TC, Biele G, Mühlemann B, Veith T, Schneider S, Beheim-Schwarzbach J, et al. Estimating infectiousness throughout SARS-CoV-2 infection course. Science. 2021; doi: 10.1126/science.abi5273 34035154 PMC9267347

[14] Robertson LS. A Critical Analysis of Peltzman’s “The Effects of Automobile Safety Regulation”. Journal of Economic Issues. 1977;11(3):587–600. doi: 10.1080/00213624.1977.11503463

[15] Greenwood J, Kircher P, Santos C, Tertilt M. An Equilibrium Model of the African HIV/AIDS Epidemic. Econometrica. 2019;87(4):1081–1113. doi: 10.3982/ECTA11530

[16] Geloso V. Masks, Seatbelts, and Peltzman Effects—AIER;. Available from: https://www.aier.org/article/masks-seatbelts-and-peltzman-effects/.

[17] Hedlund J. Risky business: safety regulations, risk compensation, and individual behavior. Injury Prevention. 2000;6(2):82–89. doi: 10.1136/ip.6.2.82 10875661 PMC1730605

[18] Trogen B, Caplan A. Risk Compensation and COVID-19 Vaccines. Annals of Internal Medicine. 2021; p. M20–8251. doi: 10.7326/M20-8251 33646837 PMC7983310

[19] Kermack WO, McKendrick AG. A contribution to the mathematical theory of epidemics. Procceedings of the Royal Society. 1927;115(772):700–721.

[20] Donsimoni JR, Glawion R, Plachter B, Wälde K. Projecting the Spread of COVID19 for Germany. German Economic Review. 2020;21:181–216. doi: 10.1007/s10273-020-2631-5 32336803 PMC7174816

[21] Dehning J, Zierenberg J, Spitzner FP, Wibral M, Neto JP, Wilczek M, et al. Inferring COVID-19 spreading rates and potential change points for case number forecasts. Science. 2020;369 (6500). doi: 10.1126/science.abb9789 32414780 PMC7239331

