# Supplementary material for: Is large-scale rapid CoV-2 testing a substitute for lockdowns?
Source: PLoS One. 2022 Mar 18;17(3):e0265207. doi: 10.1371/journal.pone.0265207 (PMC8932588; doi:10.1371/journal.pone.0265207)
Supplement: S1 Appendix — (PDF) [file pone.0265207.s001.pdf]

Appendix: Is large-scale rapid CoV-2 testing a substitute for lockdowns?

Marc Diederichs[1], René Glawion[2], Peter G. Kremsner[3,4], Timo Mitze[5], Gernot J. Müller[3,6,7], Dominik Papies[3], Felix Schulz[1], Klaus Wälde[1,6,8*]

1 Johannes Gutenberg University Mainz, Mainz, Germany
2 University of Hamburg, Hamburg, Germany
3 University of Tübingen, Tübingen, Germany
4 Centre de Recherches Médicales de Lambaréné, Lambaréné, Gabon
5 University of Southern Denmark, Odense, Denmark
6 CESifo
7 CEPR
8 IZA

* waelde@uni-mainz.de

1 Appendix

A1 The experiment

In order to appreciate the experiment under study, consider the developments in Germany prior to this experiment. German policy measures in response to the Corona pandemic are set at the state level. While policies differed somewhat across the 16 German states, all states agreed to a range of measures in response to the second wave in December 2020. In particular, non-essential shops, restaurants, and schools were closed. These measures were partly reversed in early March 2021 against the backdrop of rising infections numbers, presumably because the second wave of infections had died off by late February.

Tübingen is located in the state of Baden-Württemberg (BW, for short). Here, non-essential shops were opened on 8 March 2021, provided that the case rate in the county was below 50. Otherwise, a 'click & meet' scheme was put in place (i.e., shopping was permitted for customers with appointment). Teaching at primary schools resumed on March 15. These measures were announced on March 5 by the state government and hence implemented on short notice.

Table A1.1. Time of policy experiment

Date	Change in permitted activities
March 16, 2021	Opening of nonessential shops, outdoor dining, theaters, cinemas, etc. in Tübingen city center; official negative rapid test (”day ticket”) required
March 27, 2021	Number of day tickets for visitors from outside Tübingen county limited to 3,000 per day
April 1, 2021	Day tickets no longer available to visitors from outside Tübingen county
April 6, 2021	Outdoor dining no longer possible
April 24, 2021	Experiments ends

While regulations in Tübingen were mostly the same until then, the state government announced on March 15 that starting the next day (see table A1.1), the town of Tübingen would embark on a special experiment, centered around a large-scale rapid testing scheme, officially labeled 'Opening under Safety' ('Öffnen mit Sicherheit'). The town set up 9 testing posts where everybody could be tested with a rapid antigen test free of charge. The capacity for daily testing was 9000 and there were more than 30K tests per week [34]. 15 minutes after the test, the result would be released and in case it was negative, the subject was provided with a 'day ticket' entitling the holder to shop in non-essential stores, attend bars and restaurants (outdoors), cinemas and theaters (the OuS activities). In case the test was positive, people were asked to take a PCR test. A positive PCR test result is automatically reported to the public health office ('Gesundheitsamt'). The PCR tests form the basis for the official statistics on which our analysis is based. OuS ended on Saturday, 24 April, due to a change in the 'Bundesinfektionsschutzgesetz' adopted by the German federal parliament.

Below the level of the 16 states, Germany is subdivided into a total of 401 counties ("Landkreise" and "kreisfreie Städte"). Tübingen city (pop: 91K) is part of Tübingen county (pop: 229K). In total, there are 44 counties in BW. The experiment under study took place in Tübingen city only. Still, everyone living in Tübingen county was allowed to participate. Hence, spillovers from the city to other areas of the county may have potentially been significant. Our main analysis therefore focuses on a comparison of Tübingen county to other counties. Also, detailed data is available only at the county level.

To measure the causal impact of OuS, it is important to note that Tübingen is not exceptional in terms of fundamentals. However, it performed relatively well compared to its BW peers regarding CoV-2 case numbers (see appendix A6.2 for more background). At some point, Tübingen county was indeed enjoying the lowest case rate in all of BW. Still, there have been many counties which did similarly well during the period. The experiment taking place in Tübingen rather than elsewhere is most likely due to local idiosyncrasies and politics that are orthogonal to infection dynamics. The experiment, while approved by the state government, was devised jointly by the town's major and the Corona commissioner of Tübingen county. Both have gained prominence in national media as a result of vocal and eloquent interventions regarding the handling of the pandemic and, more importantly, because of their personalities. It seems that these personalities, rather than any special developments in Tübingen, have been causal for setting up the Tübingen experiment. It thus

comes close to a randomized control trial.

A2 Data

A2.1 General information

- Cases, cumulative cases and case rates

To avoid confusion, it is useful to remind us of definitions of (cumulative) cases and case rates. The number of SARS-CoV-2 cases reported on a day t is given by the number of new infections on day $t-1$. Cumulative cases over the previous d days on day t is given by the sum of cases from $t-1-d$ to $t-1$. The seven day case rate on day t is given by cumulative cases over the previous 7 days relative to t, divided by population size and multiplied by 100K.

- County-level data

Data on reported SARS-CoV-2 infections at the county level are taken from the Robert Koch Institute [35]. Infections are identified by PCR tests. For our empirical analysis, we use aggregate case numbers for each county and day based on the reporting date by local health authorities. Time-varying predictors are the average daily temperature and daily mobility changes for each county during the pre-treatment period until March 16, 2021. Mobility changes (in percent) based on individual mobile phone data are computed as the difference in mobility patterns between a specific date and the average value for the corresponding weekday from the same month in 2019 (pre-COVID benchmark period). To give a specific example: The mobility change for Wednesday, March 10, 2021, is calculated as the difference in the number of regional trips for this date and the average number of trips on Wednesdays in March 2019. We use data on daily temperatures from Deutscher Wetterdienst [15], and updated data on mobility changes per county and day are obtained from [37].

We further include time-constant cross-sectional predictors characterizing regional demographic structures and the regional health care system as in [33] based on data from the INKAR online database of the Federal Institute for Research on Building, Urban Affairs and Spatial Development [27]. We use the latest year available in the database, which is 2017, and rely on the following cross-sectional predictor variables: population density (population/km^2), the share of females in the population (in %), the average age of female and male population (in years), old- and young-age dependency ratios (in %), the number of medical doctors per 10,000 of population and pharmacies per 100,000 of population, the regional settlement structure (categorical dummy), and the share of highly educated population (in %).

- Community-level data

To supplement our county-level-analyses with an analysis at the city level, we obtained daily case rates for the city of Tübingen for the treatment period directly from the city's Corona Commissioner. For the pre-treatment period, we rely on weekly case rates that were provided to us by the local health authorities (https://www.kreis-tuebingen.de/17094149.html).

- SCM data and repository

Data used for our SCM-based analysis fall into four groups: Data for our main county analysis, for the Tübingen city analysis, for the stringency index (section A2.4) and some rapid test data (displayed in figure A2.1).

All of these data plus the corresponding Stata, R and matlab scripts are available in a public repository at https://figshare.com/s/87f712ecb2f9eaf044b1.

A2.2 Descriptive statistics

Table A2.2 shows descriptive statistics for variables we employ for our main analysis. The variables are measured at the county level and the underlying population is Germany without direct neighboring counties

of Tübingen (which are Böblingen, Esslingen, Reutlingen, Zollernalb, Freudenstadt and Calw). The latter are excluded from all analyses. Panel A contains all variables related to measuring the development of the pandemic. Panel B displays information on the time-varying predictors, mobility and average air temperature, and panel C shows all predictors related to the county's demographic structure and their health care coverage.

Table A2.2. Descriptive statistics

	Mean	S.D.	Min.	Max.
A: Data on reported CoV-2 cases				
Seven-day CoV-2 case notification rate per 100,000	116.53	72.60	3.74	663.76
Cumulative infections per 100,000 inhabitants since January 1st	6290.96	8803.45	387	164461
Cumulative cases over previous 7 days	232.10	309.93	2	7340
Cumulative cases over previous 14 days	448.63	589.31	7	13428
Neighbourhood (50km) seven-day case rate per 100k	116.01	58.55	0	552.48
B: Time-varying predictors				
Average mobility	-.10	.13	-.69	.73
Average temperature	3.70	4.81	-17.5	19
Stringency index	2.88	.18	2.38	3.16
C: Regional demographic structure and local health care system				
Population density (inhabitants/km^2)	535.44	705.39	36.13	4686.17
Share of females in population (in %)	50.60	.64	48.39	52.74
Average age of female population (in years)	45.88	2.12	40.70	52.12
Average age of male population (in years)	43.18	1.84	38.80	48.20
Old-age dependency ratio (persons aged 65 years and above per 100 of population aged 15-64 years)	34.39	5.49	22.40	53.98
Young-age dependency ratio (persons aged 14 years and under per 100 of population aged 15-64 years)	20.53	1.44	15.08	24.68
Medical doctors per 10,000 of population	14.62	4.42	7.33	30.48
Pharmacies per 100,000 population	27.04	4.91	18.15	51.68
Categorical variable$^{\$}$ for population density of NUTS3 region	2.60	1.05	1	4
Share of highly educated* persons in regional population (in %)	13.05	6.21	5.59	42.93

Notes: * = International Standard Classification of Education (ISCED) Level 6 and above; $^{\$}$ = included categories are 1) larger cities (kreisfreie Großstädte), 2) urban districts (städtische Kreise), 3) rural districts (ländliche Kreise mit Verdichtungsansätzen), 4) sparsely populated rural districts (dünn besiedelte ländliche Kreise).

A day-by-day overview of the number of tests and positive cases is provided in figure A2.1. On April 1, participation in the experiment was restricted to inhabitants of Tübingen county. Accordingly, we find a structural break in the data on this date with around twice as many tests being administered before (on average 2888 per day) than after the restriction (1373 daily tests). We are able to differentiate the number of positive tests into participants from Tübingen city, Tübingen county and elsewhere. We point out that the number of positive tests taken by visitors from the county after April 1 can be explained by commuting staff that was also frequently tested.

Figure A2.1. Total number of tests (left) and positive tests by origin of tested individual (right)

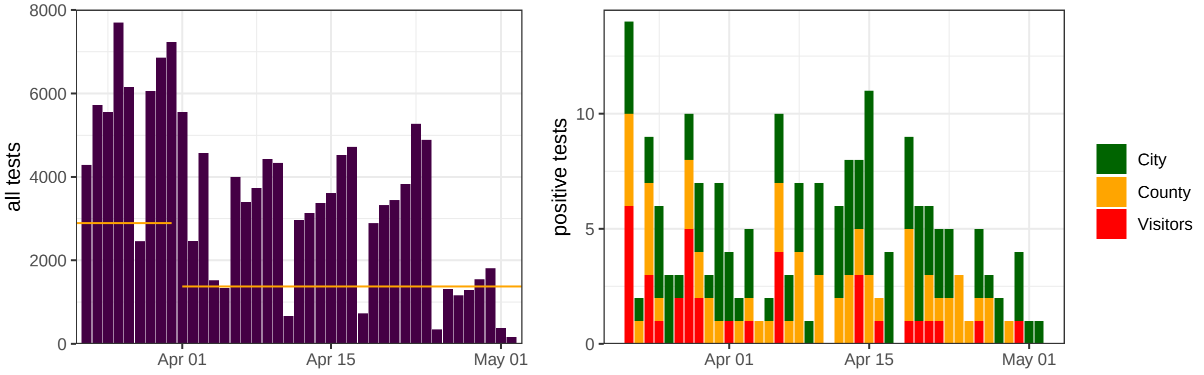

A2.3 Tübingen and its donor pool

The SCM selects control counties from a donor pool. When we compare Tübingen to all regions in the donor pool, we get a first idea about the trend of Tübingen relative to other counties and about the highest and the lowest possible treatment effect. Figure A2.2 plots case rates of Tübingen county and Tübingen city within case rates of all German counties in the left panel. The right panel shows the same two time series within case rates of all counties from Baden-Württemberg.

Figure A2.2. Seven-day case rates in Germany (left) and Baden-Württemberg (right)

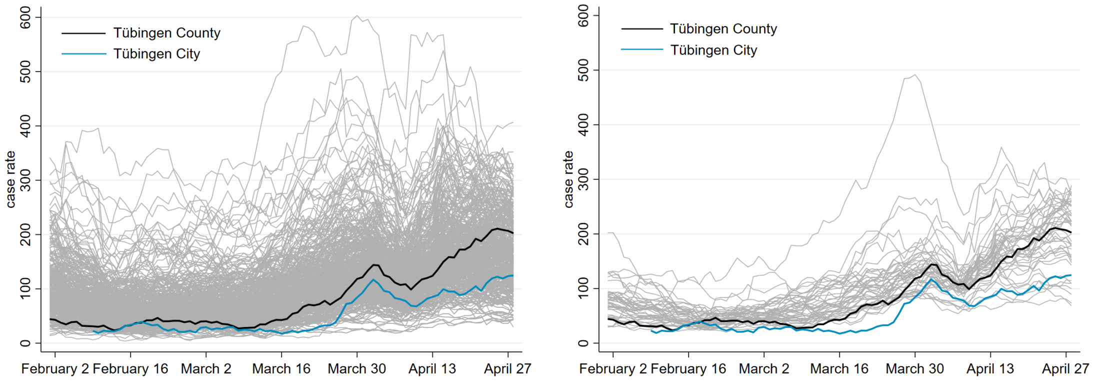

Judging the effect of OuS from these figures is difficult for many reasons. One reason is that treated and control regions start from different levels. Figure A2.3 therefore normalizes the case rate on the treatment date (16 March) in all counties to 1. One can then directly read from the resulting figure whether the growth process in the treated regions was stronger over the treatment period than in control regions.

While these figures, of course, also do not allow to draw any causal conclusions about OuS, the relative increase of Tübingen city to Tübingen county came as a surprise. While Tübingen city always has a lower incidence (level), its incidence growth is much more pronounced, both relative to Tübingen county and relative to all counties in Baden-Württemberg.

Figure A2.3. Normalized seven-day case rates in Germany (left) and Baden-Württemberg (right)

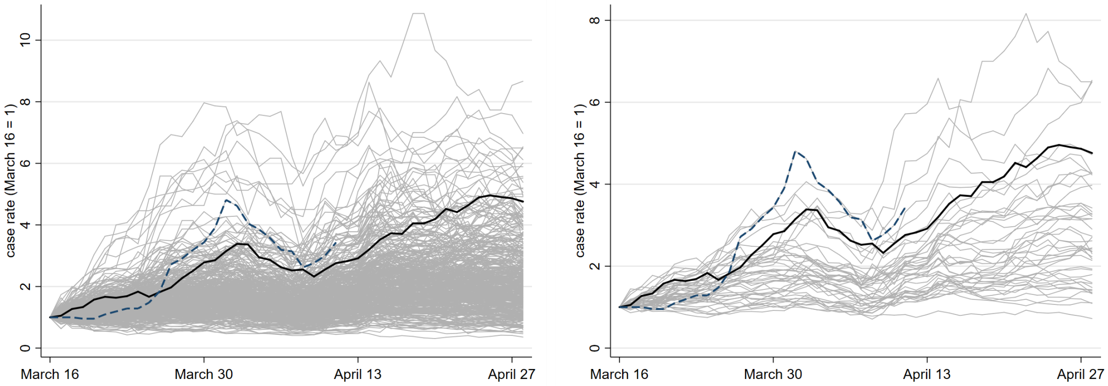

A2.4 A stringency index for German states and counties

Understanding the effects of almost anything related to the pandemic requires a detailed understanding of the institutional environment. Any region is subject to a long list of regulations that govern contacts in the private, in the public and on the workspace. In our analysis, we account for the largely decentralized policy framework enacted by German states and counties. Ideally, our synthetic control region consists of counties with a regime similar to pre-OuS conditions in Tübingen. To this end, we construct an index of the stringency of health regulations, similar to previous efforts based on an ordinal classification of measurements [25, 31]. Building on the Infas database [1] and prior experience in this field of research [33], we are able to observe differences across counties.

The available data allows us to distinguish between $k = 1...K$ domains like e.g. kindergartens, shops and restaurants. Up to 16 distinct policies are documented for each domain. In line with the other indices, we group the policies by strictness into five levels from 0 to 4. Hence, for each region i, day t and domain k, there is a vector $L_{i,t,k}$ with 16 values. The highest of these 16 values is given by $\max L_{i,t,k}$. The index $I_{i,t} \in [0, 4]$ is then calculated as daily averages of the maximum value of the $K = 23$ domains,

$$I_{i,t} = \frac{1}{K} \sum_{k=1}^{K} \max L_{i,t,k}. \tag{A2.1}$$

A first impression of the stringency of policies over time is given in figure A2.4. We plot the index I on the vertical and time on the horizontal axis. Each of the German states is represented using an individual color, Tübingen county is highlighted as the black line. As the figure reveals, policies are very homogeneous across counties. While there are 401 counties in Germany, there are rarely more than 20 different values visible at any point in time.

For our SCM, we include the index in the pre-treatment period in the predictor set. This provides us with a control group that enforced measurements of similar scope and severity prior to the experiment as Tübingen did. We cannot use the index, however, to detect other OuS projects across Germany. This is due to the focus of the Infas database on county legislature. OuS projects are in most cases restricted to single communities and in many cases not mentioned in the county legislature. This makes it impossible to point out relevant regions based on the index. We therefore proceed with an exclusion of counties based on our compilation of OuS projects across Germany in table A2.3. The source for this search is general public information. We make sure that none of these counties appears in any of our synthetic control counties.

Figure A2.4. Stringency index across all 401 German counties.

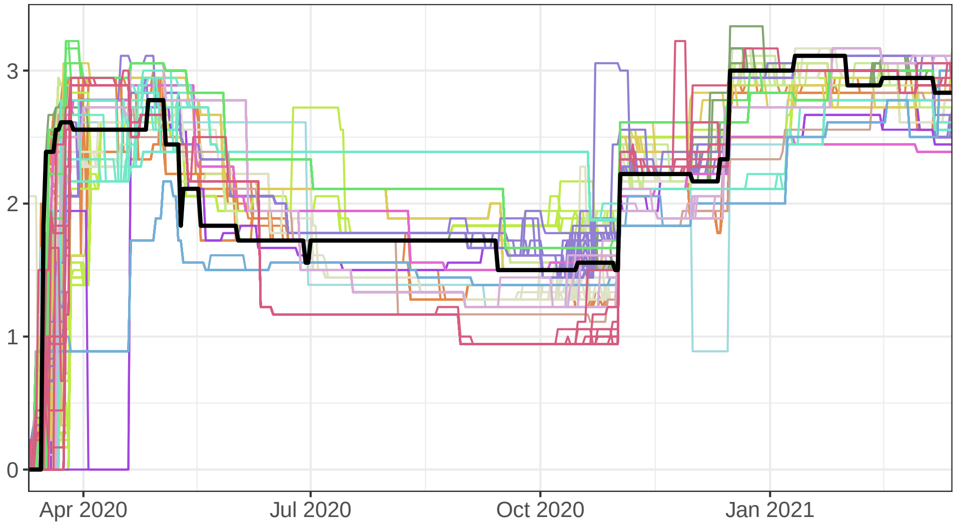

Table A2.3. Opening under Safety projects in Germany as of April 23, 2021

Community	County	Start	End	Source
Tübingen (City)	Tübingen	March 16	April 23*	tuebingen.de
Weimar (City)	Weimar (City)	March 29	March 31	tmasgff.de
Augustusburg	Mittelsachsen	April 1	April 23*	augustusburg.de
Nordhausen	Nordhausen	April 6	April 16	mdr.de
Alsfeld	Vogelsbergkreis	April 8	April 15	alsfeld.de
several	Harz	April 9	April 23*	kreis-hz.de
Baunatal	Kassel	April 12	April 23*	baunatal.de
several	Schleswig-Flensburg	April 19	ongoing	ostseefjordschlei.de
several	Rendsburg-Eckernförde	April 19	ongoing	ostseefjordschlei.de
all counties in the state of Saarland		April 6	April 23*	saarland.de

Note: All end dates marked with a * have ended due to the enactment of unitary federal restrictions on April 24 [14]

A3 Literature

There have been calls for comprehensive and large-scale testing schemes early in the pandemic [36]. In theory, it is clear that testing and quarantining can dramatically reduce the costs of an epidemic [19]. A systematic empirical assessment, however, of the benefits of widespread rapid testing based on antigen tests is still missing [24]. In the present paper, we contribute to such an assessment by studying a unique policy experiment in which widespread rapid antigen tests were coupled with opening of non-essential infrastructure. We estimate the causal effect of this intervention using the synthetic control method [5, 4, 2]. This method, SCM for short, is the vehicle for our empirical identification strategy.

SCM has been frequently used in the social sciences to study the effect of policy interventions, broadly defined, on political, social, and economic outcomes [2]. In these contexts, SCM has been shown to be a flexible and robust estimation tool. In addition, it has also been applied to COVID-related research, for instance, to study the effectiveness of lockdown measures by means of a counterfactual analysis for Sweden [8, 12] and to study the effect of shelter-in-place policies in California [21]. [33] use SCM to study the effect of face masks on SAR-CoV-2 cases in Germany. The SCM approach was also used in the interim evaluation of the Liverpool mass-scale testing project [38]. Similar to the Tübingen experiment, this pilot was centered around repeated testing of asymptomatic individuals. Those with a negative result were not allowed, however,

to participate in otherwise restricted activities. Compared to the synthetic control region, they find that large scale testing does not significantly decrease case numbers and hospitalization. In a different context, SCM allowed quantifying the impact of the Brexit referendum on economic performance in the UK [9].

A4 Findings

A4.1 Our baseline result

The synthetic twin county employed in figure 1 consists of control counties who are listed, jointly with their weights, in the main text in table 1. Table A4.4 below displays the criteria (predictors) which serve as basis for constructing the synthetic twin. Predictor values pertain to the pre-treatment period ending March 16, 2021.

Predictors can be split into groups: lagged pandemic measures (the outcome variable) and structural regional characteristics, which are expected to influence the local infection dynamics over time. As the table shows, we place a strong emphasize on lagged values of the seven-day case rate as predictor in order to ensure that Tübingen and the selected control regions follow a common pre-trend in the last two weeks before the OuS experiment stated in Tübingen. We also include an average measure for the cumulative number of SARS-CoV-2 cases in the two weeks before treatment start.

It would have been nice to include data on (UK) mutant shares. Unfortunately, they are not available at the county level. We (implicitly) capture mutant effect by focusing on pre-treatment pandemic dynamics when selecting control regions.

With regard to structural regional characteristics, we use both time-varying and time-constant predictors. As such, we use average levels for daily temperature and intra-regional mobility changes in the week prior to the treatment. The link between seasonality and infection dynamics has recently been studied [7]. Including mobility effectively controls for social interaction as a driver of local infection dynamics and also as a measure how closely people follow prevailing (lockdown) policy rules [30].

Additionally, we control for the share of females in population, average age of female population, average age of male population, old-age dependency ratio, young-age dependency ratio, medical doctors per population, pharmacies per population, categorial variable for population density of counties and share of highly educated persons in regional population as suggested in [33]. The rationale behind the inclusion of these predictors is to match Tübingen as closely as possible to its synthetic control group in terms of socio-demographic factors and factors related to the local health care system. Previous research has shown that these factors are significantly related to differences in COVID-19 incidence and death rates at the sub-national level [29].

The fit of the weighted predictor variables of the control counties from table 1 with respect to corresponding variables in Tübingen can be seen from comparing column 2 and 3 in table A4.4. Overall, there is a good fit. The population density is roughly twice as high in the synthetic control group as in Tübingen. We do not believe, however, that this is crucial for our findings. If anything, our effects are underestimated as the speed at which infection spread should be higher in more densely populated counties. The overall good fit underlines the good pre-treatment fit between the seven-day case rate development in Tübingen and its synthetic control group, as already visualized in figure 1.

Table A4.4. Balancing properties of predictor set 'baseline' for figure 1

	Treated	Synthetic
Seven-day case rate per 100k (Feb 1)	44.30	49.48
Seven-day case rate per 100k (Feb 8)	31.45	34.44
Seven-day case rate per 100k (Feb 15)	31.89	32.19
Seven-day case rate per 100k (Feb 22)	40.31	32.70
Seven-day case rate per 100k (Mar 1)	39.87	37.29
Seven-day case rate rate per 100k (Mar 8)	27.02	37.12
Seven-day case rate per 100k (Mar 15)	42.97	45.26
Cumulative cases over previous 7 days (Mar 9)	63.00	72.70
Cumulative cases over previous 14 days (Mar 15)	158.00	150.28
Average mobility (Mar 9 - Mar 15)	0.00	-0.16
Average Temperature (Mar 9 - Mar 15)	4.21	5.88
Population density	434.86	945.33
Share of females in population	51.26	51.06
Average age of female population	41.67	41.91
Average age of male population	40.03	39.98
Old-age dependency ratio	24.58	24.97
Young-age dependency ratio	20.20	19.73
Medical doctors per population	15.64	19.76
Pharmacies per population	23.48	27.42
Categorial variable for population density of NUTS3 region	2.00	1.86
Share of highly educated persons in regional population	26.47	26.09
Stringency Index	2.97	2.96
Neighborhood (50km) seven-day case rate per 100k (Feb 1)	81.64	80.99
Neighborhood (50km) seven-day case rate per 100k (Feb 8)	61.83	63.29
Neighborhood (50km) seven-day case rate per 100k (Feb 15)	51.14	49.45
Neighborhood (50km) seven-day case rate per 100k (Feb 22)	44.51	47.05
Neighborhood (50km) seven-day case rate per 100k (Mar 1)	58.36	49.55
Neighborhood (50km) seven-day case rate per 100k (Mar 8)	56.70	54.15
Neighborhood (50km) seven-day case rate per 100k (Mar 15)	70.73	72.95
RMSPE (pre-treatment)	9.90	

Note: Dates in parentheses indicate when the respective variable was measured.

A4.2 Separating the effect of opening and the effect of 'safety' (testing) in OuS

Section A5.2 presents an extended SIR model which allows us to disentangle the effect of testing from the effect of opening and to understand the effect of the Easter break. The following section shows the effect of testing while the subsequent section A4.3 focuses on the effects of the Easter break.

- Distinguishing testing from opening

We use the baseline calibration (figure A5.9) to compute the evolution of the case rate in case of no testing. We simply set the detection rate equal to zero, $\lambda^{\text{test}} = 0$, as of t=March 16 and solve the model again.

Figure A4.5 considers the period from the beginning of OuS (16 March) to its end (24 April). The left panel shows the case rate under OuS as of March 16 (black graph). The black graph captures the general pandemic dynamics, the effect of more contacts and the effect of more testing, i.e. the general pandemic plus OuS. The solid graph is the best fit of the model case rate to observed data (see top right panel in figure A5.9).

The counterfactual scenario of no testing (red graph) yields case rates that would have been observed if only opening had taken place, i.e. if only (a) the contact rate had increased in Tübingen but without (b) the identification of asymptomatic infected individuals and without (c) individuals entering quarantine. Comparing this counterfactual scenario to baseline shows that case rates would have been lower initially

Figure A4.5. The effect of testing on case rates (left) and the true pandemic state (right)

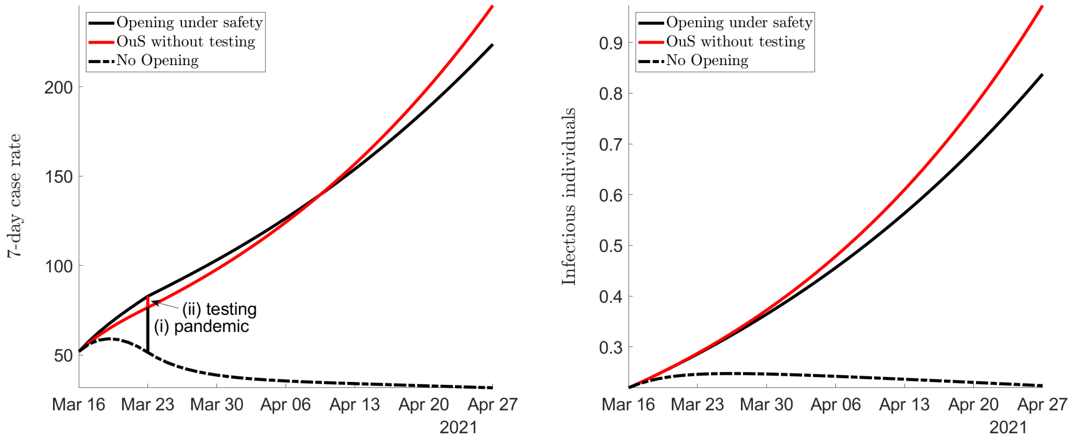

but then higher as of April 10. The part due to testing (consisting of (b) and (c) and shown as (ii) in the figure) on case rates can be positive (which is (b): more testing leads to more reporting) or negative (which is (c): more testing puts more infectious individuals into quarantine). The example highlighted in the figure therefore qualifies the statement that more testing leads to more reported cases. The figure also highlights the difference (i) between the control region and Tübingen due to the pandemic effect of OuS.

Let us now turn to the true pandemic state, defined as (the share of) individuals that are infectious (reported or not). The right panel shows that the number of infectious individuals under rapid testing (black) falls as of the moment testing starts relative to the number of infectious individuals without rapid testing (red). Testing is obviously always good for the true pandemic state but not necessarily so for the measured pandemic state (i.e. reported case rates). Quantitatively, the increase of case rates due to testing in Tübingen is very small. When we divide the black by the red graph (left panel), the maximum increase in the case rate due to rapid testing is below 7% (see bottom right panel in figure A5.9).

This analysis shows that we should correct reported case rates by the factor found in the bottom right panel in figure A5.9. We would obtain a time series for revised case rates that reflects the effect of opening only, but not the effect due to more testing. This is point (i) above. The corrected cases (i) are then the appropriate time series for Tübingen that should be compared to its control unit. Looking at these graphs as of April 10, however, the case rate is actually lower in the case of OuS. As of April 10, we therefore need to *increase* reported numbers. On April 27, case rates under OuS without the testing effect would have amounted to 226 instead of 219 under OuS. Observing a maximum initial correction of 7% and the requirement to increase case rates as of April 15, it is clear that we would reach the same conclusion about OuS in Tübingen as in our baseline analysis in figure 1.

- Increasing the testing pace

To illustrate the principle effect of testing even more clearly, consider figure A4.6. It replicates the findings just reported (OuS and OuS without testing) and asks what the effects would have been if testing had been higher by a factor of 5 (yellow curve) or even 10 (green curve).

One can see the discrepancy between what is measured (the case rates in the left panel) and the true pandemic state (in the right panel) most clearly with the green graph. When we test 'a lot' (i.e. 10 times more than what actually took place in Tübingen), the reported case rate goes up a lot (left), yet, the true pandemic state goes down strongly (right). Again, the argument that more testing leads to higher cases is confirmed, but only initially. After around 4 weeks, the number of reported cases is lower even when testing 'a lot'. As the right panel shows, the more is tested, the lower individual infections. This again stresses the discrepancy between the true state of the pandemic (right panel) and the measured state (left panel).

Figure A4.6. The effect of increasing the number of rapid tests

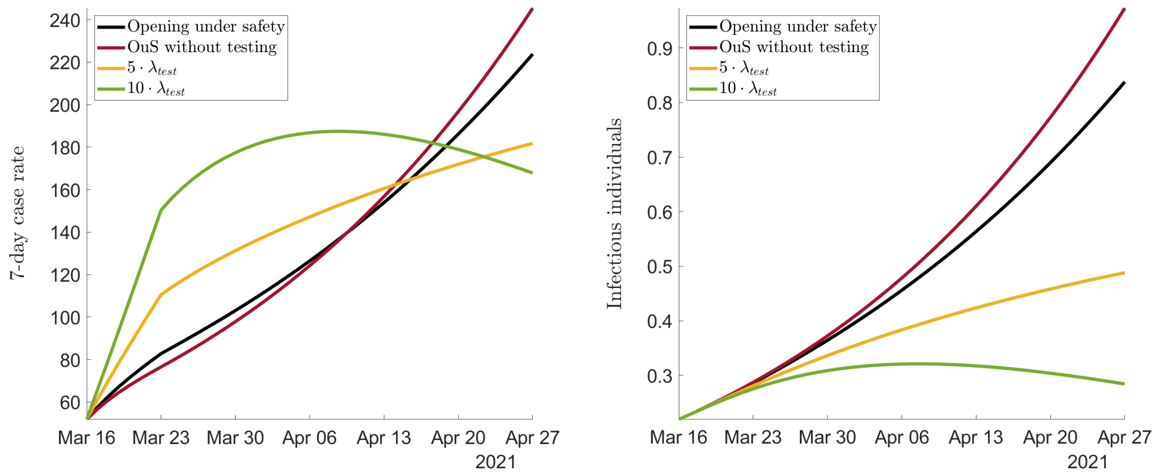

- An 'intuitive' data correction

In earlier work [16], we corrected observed case rates by the number of positive tests in rapid test centers (adjusted for a factor taking false positive tests into account). To understand why this more intuitive approach of correcting for "more cases due to more testing" is theoretically not consistent, we go back to our true pandemic state, the number of infectious individuals, shown in the right panel of figure A4.5. When we take the theoretical tradition in epidemiology, as cast into various versions of the SIR model, seriously, we can ask whether subtracting positive tests from observed cases (analysing test *rates* and case *rates* would lead to the same insight) leads to a measure which is related to the true pandemic state. Given our SIR model (A5.4) - (A5.10), reported cases are given by $I_r(t)$. When we remove the number of positive tests $\lambda^{\text{test}} I_n(t)$ from the inflow into I_r in (A5.6), we do get some corrected number of cases. It would be described by the ODE $\dot{I}_r^{corrected}(t) = (1 - x(t))\, \lambda_c^{\text{symp}} E(t) - (\eta + \rho_c)\, I_r(t)$.

This 'intuitive' correction does not lead to a measure of the true pandemic state, however, as any reference to asymptomatic cases $I_n(t)$ is missing. We therefore stick to our theoretically better founded correction displayed in figure A4.5.

A4.3 OuS and Easter break

We understand our SCM findings such that OuS increases contacts and thereby case rates, even though additional testing leads to the detection of more cases, and the Easter break leads to a temporary dip in case rates. We would now like to understand whether our extended SIR model can support this interpretation. In addition to quantifying the effect of testing, we now also take the effect of the Easter break into account. To this end, we estimate the share of individuals that do not consult medical emergency services during public holidays.

All calibrated parameters are collected in table A4.5. The table shows the contact rate a and parameters α and β from our specification of the infection rate (A5.11), the detection rate λ^{test} with which asymptomatic individuals are identified and the share x of symptomatic individuals that do not visit health centers during holidays. See the illustration of our SIR model in figure A5.8 and the full SIR model in (A5.4) - (A5.10).

Table A4.5. Calibration Results for the SEIR model

Region	a	α	β	λ^{test}	x
OuS only	.0040	4.8e-05	.9999	.0151	—
Control	.0016	.1764	6.1e-07	0	—
OuS & Easter throughout	.0029	5.9e-07	.767	.0139	.3280
OuS & before Easter	.0038	.0423	.792	.0117	—
OuS & as of Easter	.0022	1e-05	.6172	.0132	.4602

- Testing only

When we estimate the testing effect only and neglect the Easter Break, we get the first line in table A4.5 for Tübingen. The corresponding values for the control county is in the second line. These values were used above to plot figures A4.5, A4.6 and A5.9. The general findings are discussed in section 'Case rates and testing' in the main part.

- Full estimation: testing and the Easter break

Our preferred theoretical interpretation of the empirical findings in figure 1 results from calibrating one contact rate all throughout the OuS period and the Easter effect. The calibrated values are in line 3 "OuS & Easter throughout" in table A4.5. While the contact rate a does not differ strongly from the first line without the Easter break, the detection rate λ^{test} drops by around 50%. This is not surprising, however: When more individuals with symptoms do not go and visit a doctor (x is positive), the pool of non-reported individuals is larger. We therefore obtain a lower detection rate to match the same observed number of positive tests. The plot corresponding to line 3 is in figure A4.7. We see that the fit of cumulative cases (top left) and number of positive rapid tests (bottom left) are as good as they were with the earlier calibration visualized in figure A5.9.

Figure A4.7. OuS and Easter break for identical parameters

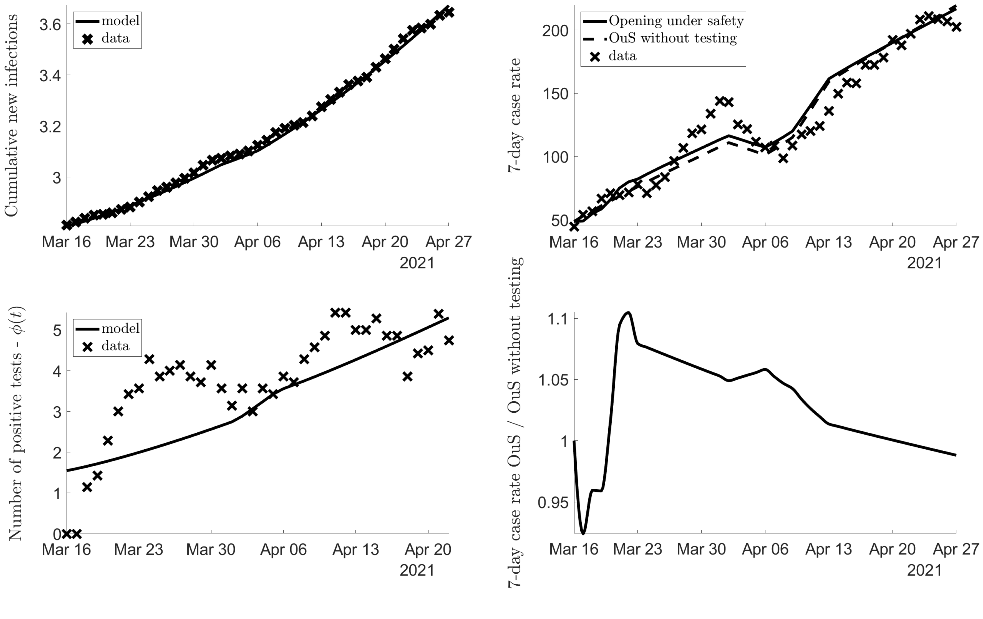

Most importantly for our view, the downturn of the case rate during the Easter break can be easily explained by a reduction of testing and reporting. Our calibration shows that $x = 32.8\%$ of individuals do not go to emergency units during public holidays but rather stay at home. This leads to a temporary drop in

case rates. (Conceptually, the drop is the same in the control region. In levels, case rates almost become identical.) After the Easter break, case rates increase again.

While the drop is smaller in the model than in the data, the model perfectly replicates the increase in case rates over the entire OuS period. Hence, we conclude that the permanent effect of OuS led to the rise in cases in Tübingen over time, the temporary dip is due to the Easter break.

When we quantify the testing effect plus the Easter Break and allow for 'before Easter' and 'as of Easter effects' on contact rates, we get the fourth and fifth line in table A4.5 for Tübingen. Note that it makes sense to distinguish these two sub-periods as visible in figure A2.1 (left). This figure clearly shows that the average number of visitors dropped to around 50% after Easter. This is perfectly consistent with the drop in the contact rate a in line 4 'OuS before Easter' to the value in line 5 'OuS as of Easter'.

The share of individuals not visiting a doctor is somewhat higher at 46.0% under this specification. The figure for this specification is almost indistinguishable from figure A4.7. We see this as further confirmation of our interpretation of the baseline findings of our SCM analysis.

A final thought experiment might further strengthen our point. Imagine, there is no testing at all during holidays. Let the OuS region have a high and the control region a low case rate just before holidays. Then, during holidays, case rates drop to zero. Regions seem to be identical in terms of reported cases. More generally speaking, the *relative* drop in case rates is the same across regions over holidays. As the difference is measured in *levels*, the difference falls.

In the background, however, the pandemic continues, it is simply not measured. When holidays are over, visits at GPs and testing resume and the difference between the treated and the control region is again visible in the data. The fundamental and crucial difference in the regions consists in the difference in the contact rates.

A5 Methods

A5.1 The synthetic control method

The synthetic control method (SCM) is by now a well established strategy to measure the *treatment effect* of specific policy measures (see Section A3 for references). It has proven to be a useful tool in the context of the Covid-19 pandemic to study e.g. the effect of making face masks mandatory or to quantify the effect of lockdown measures [33, 8].

Here we provide the details regarding SCM that are relevant for our analysis. First, we set up the *donor pool*: it includes 400 Germany counties ("Landkreise" und "kreisfreie Städte"). 34 of these are located in BW and hence in the same state as Tübingen county. We exclude counties adjacent to Tübingen county (listed in appendix A2.2) from the donor pool given a high likelihood of spillovers. We consider alternative donor pools in order to corroborate our results.

Second, we construct a *synthetic control unit* as a weighted average across the counties in the donor pool. Note that the number of counties with non-negligible weight is not restricted by our procedure and may vary across specifications. The weights are selected on the basis of a minimum distance approach.

Specifically, we target a set of *predictor variables* for Tübingen county in the pre-treatment period (that is, before March 16) in order to determine county weights. Predictors consist of pandemic measures and non-pandemic measures. The choice of predictor variables is driven (partly by their availability and) mainly by the desire to identify comparable counties based on fundamental determinants driving the outcome variable. The predictor set includes observations for the *outcome variable* (infection rate). In an ideal world, one would include those variables as predictors which determine the evolution of the pandemic in a county. As these ideal predictors are not available, regional characteristics and lagged outcome variables serve as proxies for the latent true variables. As pandemic-independent variables, we include both population as well as district characteristics in our predictor set. To assure that the control group consists of counties with a similar pandemic situation prior to the treatment, we also take pandemic-dependent variables into account. They include case rates prior to treatment and case rates of neighboring counties.

SCM chooses the weights on the counties in the donor pool by minimizing the 'root mean square prediction error' (RMSPE) which quantifies the distance of the (weighted sum of) comparison counties to Tübingen prior to treatment. Doing so, the control unit resembles Tübingen in terms of these variables as closely as

possible. In this way, we ensure that pre-treatment differences in trends of the outcome variable are equalized. Table A4.4 lists all predictor variables. They include all socio-economic characteristics that are a) available at the county level and b) may matter for infection dynamics. In addition, we include weekly averages for infection rates in the six weeks prior to treatment.

Formally, let $\mathbf{x_1}$ denote the $(k \times 1)$ vector of predictor varibles in Tübingen and let $\mathbf{X_0}$ denote a $(k \times n)$ matrix with observations in the counties included in the donor pool consisting of n counties. Let $\mathbf{w}$ denote a $(n \times 1)$ vector of country weights w_j, $j = 1, \ldots, n$. Then, the control unit is defined by a $\mathbf{w}^*$ which minimizes the mean squared error

$$(\mathbf{x_1} - \mathbf{X_0 w})'\mathbf{V}(\mathbf{x_1} - \mathbf{X_0 w}) \ , \tag{A5.2}$$

subject to $w_j >= 0$ for $j = 1, \ldots, n$ and $\sum_{j=1}^{n} w_j = 1$. In this expression, $\mathbf{V}$ is a $(k \times k)$ symmetric and positive semidefinite matrix. Here, $\mathbf{V}$ is a weighting matrix assigning different relevance to the characteristics in $\mathbf{x_1}$ and $\mathbf{X_0}$. Although the matching approach is valid for any choice of $\mathbf{V}$, it affects the weighted mean squared error of the estimator [4]. We choose a diagonal $\mathbf{V}$ matrix such that the mean squared prediction error of the outcome variable (and the covariates) is minimized for the pre-treatment period [5, 4].

We conduct all SCM estimations in STATA using the SYNTH [3] and SYNTH_RUNNER [23] packages. Our implementation follows largely [33].

Confidence intervals (CIs) are calculated from one-sided pseudo p-values obtained on the basis of comprehensive placebo-in-space tests. The latter tests calculate pseudo-treatment effects for all counties in the donor pool assuming that they, rather than Tübingen, were treated with OuS on March 16, 2021. We calculate one-sided pseudo p-values as the share of placebo-treatment effects that are larger than the observed treatment effects for treated counties. We thereby indicate the probability that the increase in the outcome variable was observed by chance given the distribution of pseudo-treatment effects in the donor pool.

To account for differences in pre-treatment match quality of the pseudo-treatment effects, only donors with a good fit in the pre-treatment period are considered for inference. Specifically, we do not include placebo effects in the pool for inference if the match quality of the control region, measured in terms of the pre-treatment root mean squared prediction error (RMSPE), is greater than 10 times the match quality of the treated unit [11]. Based on the obtained pseudo p-values we calculate confidence intervals as described in [6].

A5.2 A quantitative SIR model for Opening under Safety

To understand the effects of opening under safety (OuS), we start from an extended description of a pandemic illustrated in figure A5.8. Simpler models of this type have been employed e.g. by [33], [18] or [13].

- A SIR model with testing and public holidays

Susceptible individuals $S(t)$ can be infected after which they are exposed $E(t)$. Exposed individuals can turn infectious and reported, $I_r(t)$, or infectious but not reported, $I_n(t)$. We assume that ('standard', i.e. non-rapid) tests are undertaken only for the $E(t) - I_r(t)$-flow, i.e. if individuals visit a doctor and display symptoms related to Covid-19. All reported infections are therefore symptomatic infections (Covid-19 cases). Tests employed in this case are PCR tests. Individuals can recover, $R(t)$, or enter a hospital $H(t)$. They can recover from hospital or die, $D(t)$. The flows between the different states are illustrated in figure A5.8.

This figure extends earlier SIR models in two ways that are crucial to understanding OuS in Tübingen: the testing channel and public holidays. As the dashed rectangle is intended to show, rapid testing is applied to all individuals at rate $\lambda^{\text{rapid testing}}$ that are not known to be infections (I_r), in hospital (H) or dead (D). Some rapid tests are negative (at rate $\lambda^{\text{negative}}$) and individuals stay in the state in which they were before being tested. For some individuals, however, rapid testing leads to a flow from asymptomatic, i.e. non-reported infectious individuals (I_n), to reported infectious individuals (I_r). Only non-reported infectious individuals can be detected to be infectious. This flow, visible via the rate λ^{test} in the figure and in equations (A5.6) and (A5.7) below, takes place only from I_n to reported infectious I_r. We call it the detection rate. This flow is the big promise of OuS. Rapid negative tests do not imply any flows and are therefore not visible in the equations below. Only the net flow is visible in the equations as $\lambda^{\text{test}} = \lambda^{\text{rapid testing}} - \lambda^{\text{negative}}$. The testing rate $\lambda^{\text{rapid testing}}$ describes the rate with which OuS participants are tested in Tübingen. The rate $\lambda^{\text{negative}}$ determines flows due to negative rapid tests and the detection rate λ^{test} is the residual.

Figure A5.8. A SEIR model for opening under safety and the effect of rapid testing

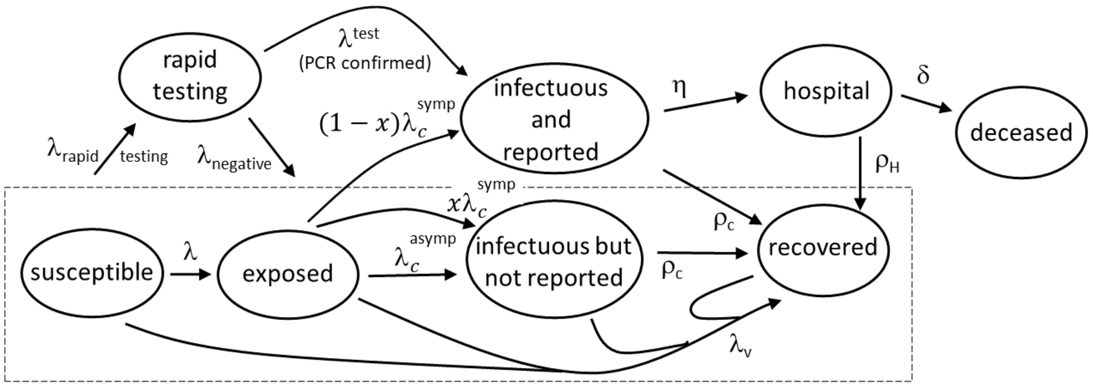

The second extension captures the effect of public holidays at Easter on the number of reported infections. During public holidays, a share $x(t)$ of individuals that feel sick stay at home. A share $1 - x(t)$ goes to emergency care. This is visible as $(1 - x(t))\,\lambda_c^{\text{symp}}$ in (A5.6) and as $(\lambda_c^{\text{asym}} + x(t)\lambda_c^{\text{symp}})\,E(t)$ in (A5.7) below. Figure A5.8 also displays this redirected flow $x\lambda_c^{\text{symp}}$. The idea behind $x(t)$ is simply that some individuals with symptoms would go to a GP during workdays but do not do so during public holidays. When public holidays are over, symptoms are weak or gone, they also do not go to the GP on the next workday. A case that would have been (tested and) counted on workdays is not counted during holidays. To capture the effects of the Easter break from Friday to Monday, we set

$$x\,(t) = \left\{ \begin{array}{c} x \\ 0 \end{array} \right\} \text{ for } t \in \left\{ \begin{array}{c} \text{April 2 to (and including) April 5} \\ \text{otherwise} \end{array} \right\} \tag{A5.3}$$

The differential equation system resulting from the underlying continuous time Markov chain (see [26] for more background) reads

$$\dot{S}(t) = -\left(\lambda(t) + \lambda_v\right) S\,(t) \tag{A5.4}$$

$$\dot{E}\,(t) = \lambda(t)S\,(t) - \left(\lambda_c^{\text{asym}} + \lambda_c^{\text{symp}} + \lambda_v\right) E\,(t) \tag{A5.5}$$

$$\dot{I}_r(t) = \left(1 - x(t)\right) \lambda_c^{\text{symp}} E(t) + \lambda^{\text{test}} I_n(t) - \left(\eta + \rho_c\right) I_r(t) \tag{A5.6}$$

$$\dot{I}_n(t) = \left(\lambda_c^{\text{asym}} + x(t)\lambda_c^{\text{symp}}\right) E(t) - (\rho_c + \lambda^{\text{test}})I_n(t) \tag{A5.7}$$

$$\dot{H}(t) = \eta I_r(t) - \left(\rho_H + \delta\right) H(t) \tag{A5.8}$$

$$\dot{R}(t) = \rho_c \left[I_r(t) + I_n(t)\right] + \rho_H H(t) + \lambda_v \left[S\,(t) + E\,(t) + I_n\,(t)\right] \tag{A5.9}$$

$$\dot{D}(t) = \delta H(t) \tag{A5.10}$$

Some of our transition rates are functions of states of the system. The rate of getting infected is given by $\lambda(t) = ap\,(I_n)\,\pi$, where a is the rate with which an individual meets other individuals, $p\,(I_n)$ is the probability that this individual gets in contact with a person being infectious and π is the probability that this leads to an own infection. In terms of an example, a is governed by how socially oriented a person is and by contact restrictions, $p\,(I_n)$ follows from the epidemic state and π is a function of the virus. As two examples, the UK-mutant or the delta-variant have a higher π than the original one.

The share of infectious individuals in all individuals one can meet in our model is given by $I_n/\,(S + E + I + R)$. In other words, we assume that there are no contacts with individuals in state I_r (they are in perfect quarantine), with individuals in hospital or with deceased individuals. Allowing for some non-linearity in arrival rates in the spirit of [18], we specify the infection rate as

$$\lambda(t) = aI_n(t)^{\beta}\left[S(t) + E(t) + I_n(t) + R(t)\right]^{-\alpha}\pi. \tag{A5.11}$$

- The effect of rapid testing

Now imagine the model is solved numerically. How do we study the effect of rapid testing on the pandemic? Note that the flow (not the rate) from I_n to I_r per unit of time is

$$\phi(t) \equiv \lambda^{\text{test}} I_n(t). \tag{A5.12}$$

This flow is observed and illustrated in the right panel of figure A2.1. Data provides us with the number of (PCR confirmed) positive rapid tests per week or day (or any other unit of time). Let us call this number pos(t). If we choose one unit of time in our SIR model to equal one day, then we can equate $\phi(t)$ with pos(t) for any day t. Computing a model solution with $\phi(t) = 0$ then shows, how the pandemic would have evolved in the absence of rapid testing. The difference to $\phi(t)$ =pos(t) provides a quantitative measure of the positive effects of rapid testing.

- Calibrating the model

The calibration of the model follows earlier work [18, 33]. We would like to match seven-day case rates, cumulative cases and the number of positive PCR-confirmed positive tests. For the initial values of H and D we use the reported data for Tübingen. The control group consists of weighted values according to Table 1. For the currently infected persons we used the number of reported infections over the last 18 days and distribute them evenly across the states I_r and I_n. We approximate the initial value of E by a backward looking measure adding the number of reported infections over the last 4 days. The number of recovered people is given by subtracting all states from N. We normalize population size to 100 and express all numbers as shares. Table A5.6 presents the exact values we use.

Table A5.6. Initial conditions for the SEIR model in %

Region	$S(0)$	$E(0)$	$I_r(0)$	$I_n(0)$	$H(0)$	$D(0)$	$R(0)$
Tübingen	97.19	0.04	0.09	0.09	0.0425	0.16	2.39
Control	97.66	0.04	0.09	0.09	0.072	0.16	1.89

We calibrate the parameter vector $\theta = \{a, \alpha, \beta, \eta, \rho_c, \rho_H, \lambda^{\text{test}}\}$ by fixing some parameters according to values taken from the literature, and by choosing $\theta = \{a, \alpha, \beta, \lambda^{\text{test}}, x\}$ such as to minimize the Euclidean distance between the reported values and the corresponding predictions of the model. Formally,

$$\min_{\theta} \left(\Phi(T) - \Phi^{\text{data}}(T)\right)^2 + \sum_{t=1}^{T} \left\{ \left(I_{r,t}^{\text{ever}} - I_{r,t}^{\text{ever,data}}\right)^2 + \left(i_{7,t} - i_{7,t}^{\text{data}}\right)^2 \right\}. \tag{A5.13}$$

In terms of timing, $t = 1$ denotes March 16, 2021 and $t = T$ is April 28, 2021. The number of positive (PCR confirmed) rapid tests at T is given by the integral over the flow, $\Phi(T) = \int_t^T \phi(s)ds$. Similarly for the number of cumulative reported cases: Starting from our ODE system (A5.4)-(A5.10), numerical consistency suggests to add a differential equation which counts all reported cases $\dot{I}_r^{\text{ever}}(t) = \lambda_c^{\text{symp}} E(t) + \lambda^{\text{test}} I_n(t)$. We solve this ODE jointly with the above system. Given the trajectory for $I_r^{\text{ever}}(t)$, we can use it to calculate the 7-day case rate by differencing the time series:

$$i_{7,t} = (I_{r,t}^{\text{ever}} - I_{r,t-7}^{\text{ever}}) \cdot 1000. \tag{A5.14}$$

Since 7-day case rates are normalized per 100,000 inhabitants and we chose our population size as $N = 100$, we multiply the rate by 1,000 to scale the model rate accordingly to the reported rates. The 7-day case rate, $i_{7,t}^{\text{data}}$, corresponds to the observed data.

We fix the remaining parameters by choosing $\pi = 1$ (which simply means that our estimate of the contact rate a is a joint estimate of $a\pi$), a vaccination rate λ_v of 2% per week, and $\lambda_c^{\text{symp}} = .22$ and $\lambda_c^{\text{asymp}} = .18$, which corresponds to a mean average incubation time of 5 days for both symptomatic and asymptomatic infections. We set the death rate to $\delta = .0268$ and $\eta = .1$, $\rho_c = 1/14$, $\rho_h = 1/21$. Table A5.7 summarizes the fixed parameter values, table A4.5 presents the outcome of solving the minimization procedure in (A5.13).

Table A5.7. Fixed parameter values.

N	η	ρ_c	ρ_H	π	λ_c^{asym}	λ_c^{symp}	δ
100	.1	1/14	1/21	1	.18	.22	.0268

In our earlier version [16], we had set $\lambda_c^{\text{symp}} = \lambda_c^{\text{asymp}} = .2$ which implies an identical incubation time of 5 days for both types of infections. In this new version, we ran a grid search for one arrival rate between 0.15 and 0.3 such that the average of the arrival rates remains 0.2 (and therefore the average incubation time remains 5 days). The pair in table A5.7 is the one with the lowest sum of our minimization procedure in (A5.13).

- Calibration results

We start with a specification that ignores the Easter effect, i.e. for $x = 0$. Figure A5.9 provides an overview of the targeted data (cumulative cases, seven-day case rate and number of positive tests), the model fit and the ratio of case rates in the case of testing to the case of no testing. Calibrated parameter values for Tübingen and its control county are in line 1 ("OuS only") and line 2 ("Control") of table A4.5.

Figure A5.9. Data and paths of calibrated model and testing-no-testing ratio

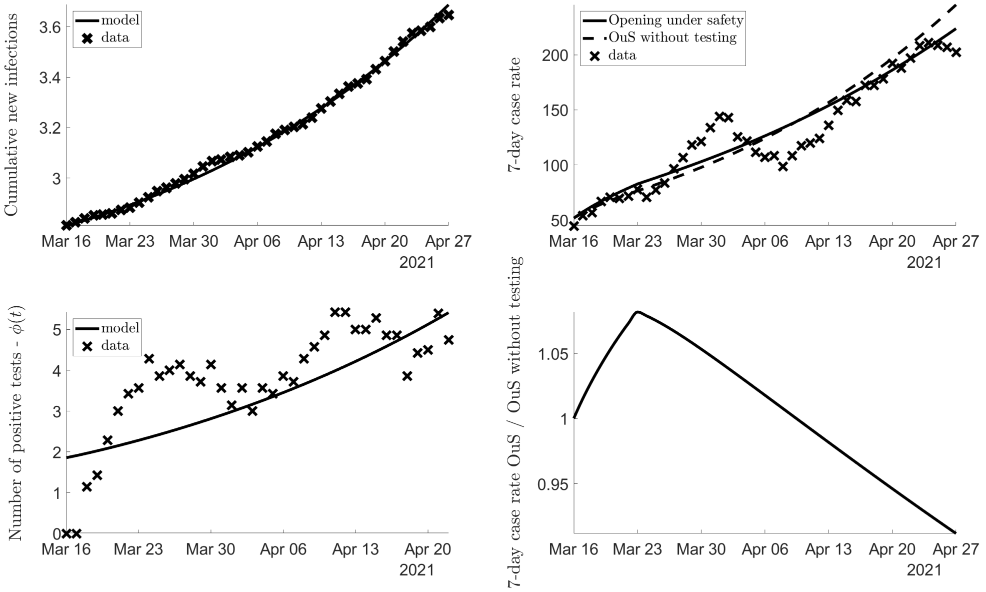

The top left panel of figure A5.9 shows cumulative new cases since 16th March, 2021. As the vertical axis shows, just below 2.8 percent of the population were infected on that date. The right panel shows the fit in terms of case rates. The crosses 'data' show the case rate as reported by health authorities. Comparing top left to top right shows that the model captures cumulative cases very well, but case rates to a lesser extent. The latter is not surprising as case rates or daily cases are much more volatile than cumulative cases. Yet, the overall tendency over the entire period of the experiment is matched perfectly for both variables. We can therefore confidently derive insights concerning the effect of testing from this calibration. Section A4.3 will explain the volatility over Easter by reduced testing and reporting.

The main purpose of employing this SIR variant consists in separating the effects of opening from the effects of testing. To obtain reliable results, the model needs to capture the total number of positive tests as well. This is achieved by estimating the detection rate λ^{test}. The lower left panel shows that this also works sufficiently well. Again, the model does not capture short-run fluctuations. This, however, is of no importance for the overall trend and our overall finding. We referred to the lower right panel in section A4.2.

A6 Discussion of donor pool

The a-priori choice of the set of counties from which SCM can choose comparison counties affects the composition of the synthetic twin of Tübingen. It is therefore crucial to understand the importance of the choice of the donor pool for the final result.

A6.1 'Leave one out'

Having found our baseline specification for figure 1, we now proceed and check the robustness of the finding by evaluating the importance of individual counties in the synthetic twin of Tübingen. These counties are shown in table 1.

Figure A6.10. Leave one out for figure 1

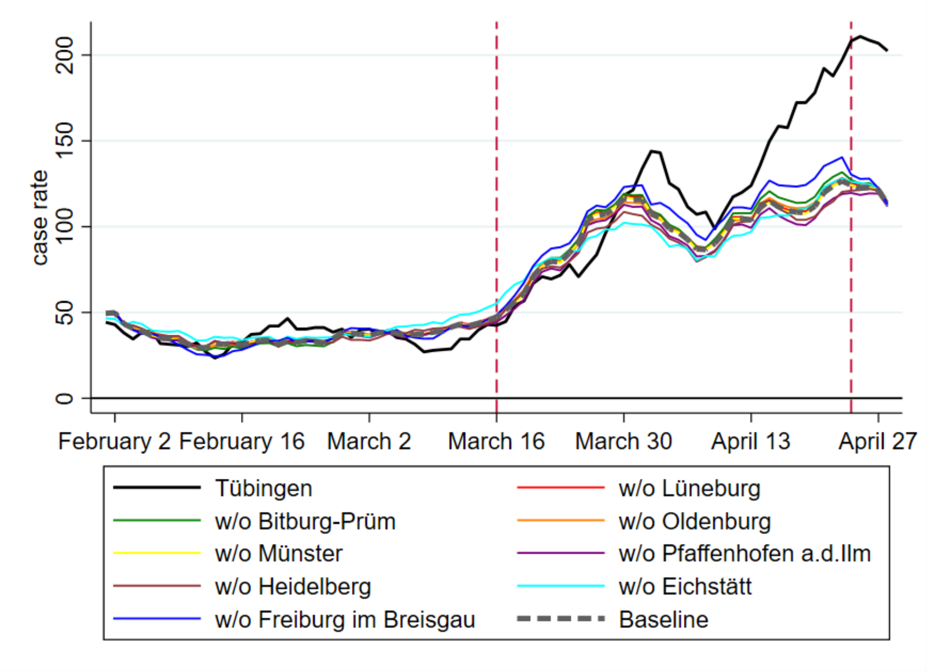

One standard approach to do this consists in removing each county shown in table 1, one by one, from the donor pool. Figure A6.10 shows the pre-treatment fit and the post-treatment effect of OuS under these adjusted donor pools. As the figure impressively shows, excluding any individual county from the donor pool does not affect our overall finding in any meaningful way. This robustness check strongly confirms our baseline finding.

A6.2 Donor pool Baden-Württemberg only

The least restrictive donor pool consists of all German counties, excluding neighboring counties of Tübingen to avoid spillover effects. As can be seen in the left panel of figure A2.2, Tübingen county is at the lower end of the German case rate distribution before March 16. However, there are many other counties with case rates slightly above or below the case rate of Tübingen that can potentially serve as synthetic control group. For these reasons, our baseline specification works with this donor pool Germany.

The donor pool Baden-Württemberg (BW) is, by construction, more restrictive. The right panel of figure A2.2 shows the case rate of Tübingen county within the BW donor pool. Tübingen sometimes has the lowest case rate, especially shortly before the beginning of OuS on 16 March. Afterwards, it moved into the middle range. For these reasons, finding a suitable control group is difficult. This issue becomes even more prominent when discussing Tübingen city later on.

Table A6.8. Control counties and their weights for figure A6.11

Name	Weight
SK Heidelberg	0.40
LK Biberach	0.24
SK Freiburg i.Breisgau	0.23
LK Ostalbkreis	0.11
LK Tuttlingen	0.01

Nevertheless and for robustness checks, we now focus on a donor pool consisting of counties from Baden-Württemberg (BW) only. When we run SCM with a donor pool consisting of all BW counties (apart from neighbours), we obtain 5 control counties as seen in table A6.8. The corresponding predictor balance is in table A6.9 which also shows our predictor set 'baseline' in the first column.

Turning to results, the left panel of figure A6.11 shows the case rate in Tübingen (in black) and in the synthetic control county (dashed line). The right panel shows the difference between the case rates in Tübingen and in the control county. When we compare the overall outcome as visible in this figure with our baseline result in figure 1, we conclude that the difference between treatment and control is somewhat smaller in figure A6.11.

Figure A6.11. Case rates for donor pool Baden-Württemberg

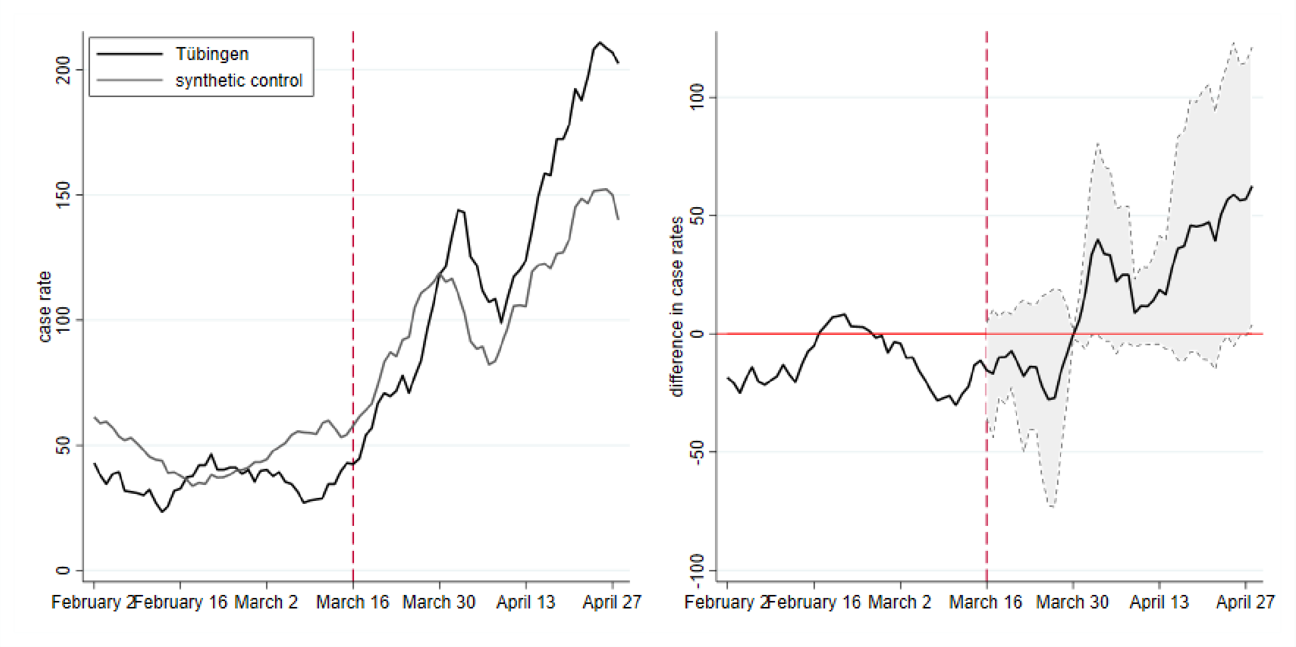

When we look at the confidence intervall, we even see that the difference between Tübingen county and its synthetic twin from Baden-Württemberg is not significant. Nevertheless, the trend in the difference (right panel) is clearly positive and rising as of end of March. We do not put too much emphasis on this finding as the pre-treatment fit with donor pool Baden-Württemberg is not as good as in our baseline scenario. This finding therefore does not affect our overall conclusion, it rather confirms our baseline finding.

Table A6.9. Balancing properties of predictor set 'baseline' for figure A6.11

	Treated	Synthetic
Seven-day case rate per 100k (Feb 1)	44.30	60.78
Seven-day case rate per 100k (Feb 8)	31.45	53.33
Seven-day case rate per 100k (Feb 15)	31.89	39.25
Seven-day case rate per 100k (Feb 22)	40.31	37.12
Seven-day case rate per 100k (Mar 1)	39.87	44.11
Seven-day case rate per 100k (Mar 8)	27.02	55.17
Seven-day case rate per 100k (Mar 15)	42.97	54.93
Cumulative cases over previous 7 days (Mar 9)	63.00	110.45
Cumulative cases over previous 14 days (Mar 15)	158.00	223.74
Average mobility (Mar 9 - Mar 15)	0.00	-0.09
Average Temperature (Mar 9 - Mar 15)	4.21	6.34
Population density	434.86	992.31
Share of females in population	51.26	51.15
Average age of female population	41.67	41.74
Average age of male population	40.03	39.77
Old-age dependency ratio	24.58	24.99
Young-age dependency ratio	20.20	19.33
Medical doctors per population	15.64	21.14
Pharmacies per population	23.48	27.61
Categorial variable for population density of NUTS3 region	2.00	1.61
Share of highly educated persons in regional population	26.47	29.29
Stringency Index	2.97	2.97
Neighborhood (50km) seven-day case rate per 100k (Feb 1)	81.64	89.76
Neighborhood (50km) seven-day case rate per 100k (Feb 8)	61.83	70.10
Neighborhood (50km) seven-day case rate per 100k (Feb 15)	51.14	51.46
Neighborhood (50km) seven-day case rate per 100k (Feb 22)	44.51	48.70
Neighborhood (50km) seven-day case rate per 100k (Mar 1)	58.36	51.36
Neighborhood (50km) seven-day case rate per 100k (Mar 8)	56.70	61.76
Neighborhood (50km) seven-day case rate per 100k (Mar 15)	70.73	77.87
RMSPE (pre-treatment)	19.57	

Note: Dates in parentheses indicate when the respective variable was measured.

A7 Discussion of pandemic measure

An alternative measure of the pandemic state consists in normalized cumulative cases since, say, January 1st, 2021. Such a measure does not look at a (normalized) moving sum (like the seven-day case rate) but simply adds up the number of infections over time. It is therefore a measure with a much longer "memory". An infection that occurred at any point since January 1st is always counted. Case rates "forget" cases that are older than, usually, 7 days. Cumulative cases are also normalized per 100K inhabitants to allow for a meaningful comparison across counties of different population size. We let then the SCM search for an appropriate comparison group for this dependent variable and compare the evolution of infections over time. Table A7.11 shows the fit between Tübingen and the synthetic twin city (table A7.10) for cumulative infections as dependent variable.

As the black and dashed curve before treatment on March 16 in figure A7.12 show, the fit between Tübingen and its synthetic twin county is almost perfect here. The better fit compared to case rates is not surprising as adding up infections since some starting date (1 January 2021 here) implies a smoother time series than adding infections over the previous 7 days. Table A7.11 shows the details of the fit.

What is much more important for our question, however, is the confirmation of our baseline result with this alternative measure. Tübingen county experiences an increase in cases relative to its synthetic control county.

Table A7.10. Control counties and their weights for figure A7.12

Name	Weight
LK Eichstätt	0.19
SK Münster	0.18
SK Freiburg i.Breisgau	0.15
SK Heidelberg	0.13
LK Lüneburg	0.12
LK Bitburg-Prüm	0.01
SK Trier	0.06
SK Rostock	0.04
SK Oldenburg	0.04

Figure A7.12. Cumulative cases per 100,000 since January 1st

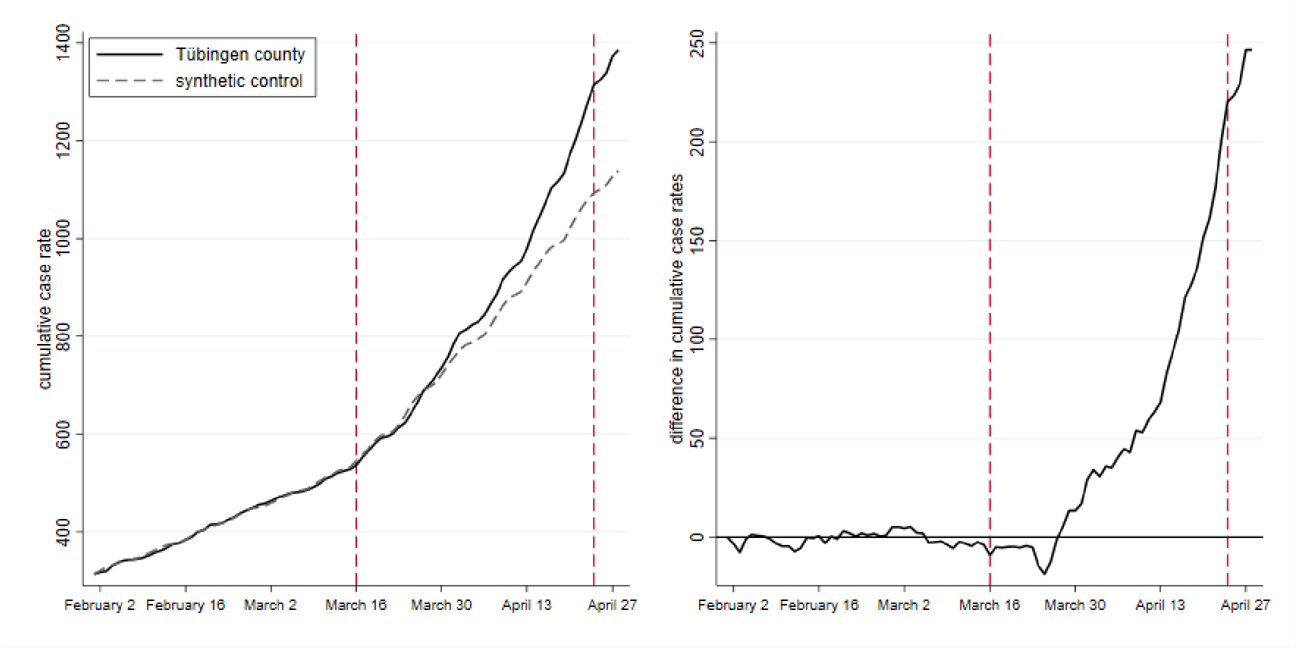

Table A7.11. Balancing properties of predictor set 'baseline' for figure A7.12

	Treated	Synthetic
Cumulative cases per 100k (Feb 1)	313.61	313.68
Cumulative cases per 100k (Feb 8)	344.62	345.59
Cumulative cases per 100k (Feb 15)	377.40	377.87
Cumulative cases per 100k (Feb 22)	417.71	417.36
Cumulative cases per 100k (Mar 1)	458.02	452.98
Cumulative cases per 100k (Mar 8)	485.93	488.05
Cumulative cases per 100k (Mar 15)	528.45	532.28
Seven-day case rate per 100k (Mar 15)	42.97	42.91
Average mobility (March 9 - March 15)	0.00	-0.17
Average Temperature (March 9 - March 15)	4.21	5.54
Population density	434.86	798.44
Share of females in population	51.26	51.05
Average age of female population	41.67	42.49
Average age of male population	40.03	40.37
Old-age dependency ratio	24.58	26.19
Young-age dependency ratio	20.20	19.56
Medical doctors per population	15.64	18.60
Pharmacies per population	23.48	27.48
Categorial variable for population density of NUTS3 region	2.00	2.04
Share of highly educated persons in regional population	26.47	23.83
Stringency Index	2.97	2.90
Neighborhood (50km) seven-day case rate per 100k (Feb 1)	81.64	79.27
Neighborhood (50km) seven-day case rate per 100k (Feb 8)	61.83	64.92
Neighborhood (50km) seven-day case rate per 100k (Feb 15)	51.14	52.00
Neighborhood (50km) seven-day case rate per 100k (Feb 22)	44.51	54.04
Neighborhood (50km) seven-day case rate per 100k (Mar 1)	58.36	53.47
Neighborhood (50km) seven-day case rate per 100k (Mar 8)	56.70	53.14
Neighborhood (50km) seven-day case rate per 100k (Mar 15)	70.73	69.14
RMSPE (pre-treatment)	4.96	

Note: Dates in parentheses indicate when the respective variable was measured.

A8 Discussion of predictor set

The predictor set is obviously important for the choice by SCM of control counties and their weights. Counties can be compared by fundamental characteristics like population density, educational background and medical services or by more pandemic-related features such as case rates prior to treatment.

As discussed in section 'Predictor sets and donor pool', we have three basic predictor sets: predictor set 'baseline' (table A4.4), predictor set 1 (table A8.13) and predictor set 2 (table A8.15). Predictor set 'baseline' is also used for table A6.9 and table A7.11. In table A7.11, case rates are replaced by cumulative cases as our pandemic measure was changed to cumulative cases. A special predictor set 'community' is needed when we inquire into Tübingen city. See section 'Does Tübingen city differ from Tübingen county?' for details.

Our baseline predictor set includes the pandemic measure of Tübingen county and its neighbours over a period of six weeks (i.e. February 1 to March 15 in table A4.4) to ensure that the pandemic measure in the control group evolved in a similar way to the pandemic measure in Tübingen. We chose the matching period to be as long as the prediction period. One could argue however, that a good match on the pandemic measure dating back multiple weeks has no predictive power for the development of the pandemic and that a the pandemic measure should be included at a higher frequency to ensure a very good pre treatment fit. This idea is captured by predictor set 1 and 2 and visible e.g. in table A8.13 with daily case rates from March 1 to March 15.

A8.1 Full donor pool and predictor set 1

We now display the results shown in our central robustness figure 2 in the main part in more detail. As figure A8.13 shows, including pre-treatment measures of the pandemic at higher frequency does not change the basic finding, even though the members of the synthetic control county change.

Figure A8.13. Seven-day case rate with predictor set 1

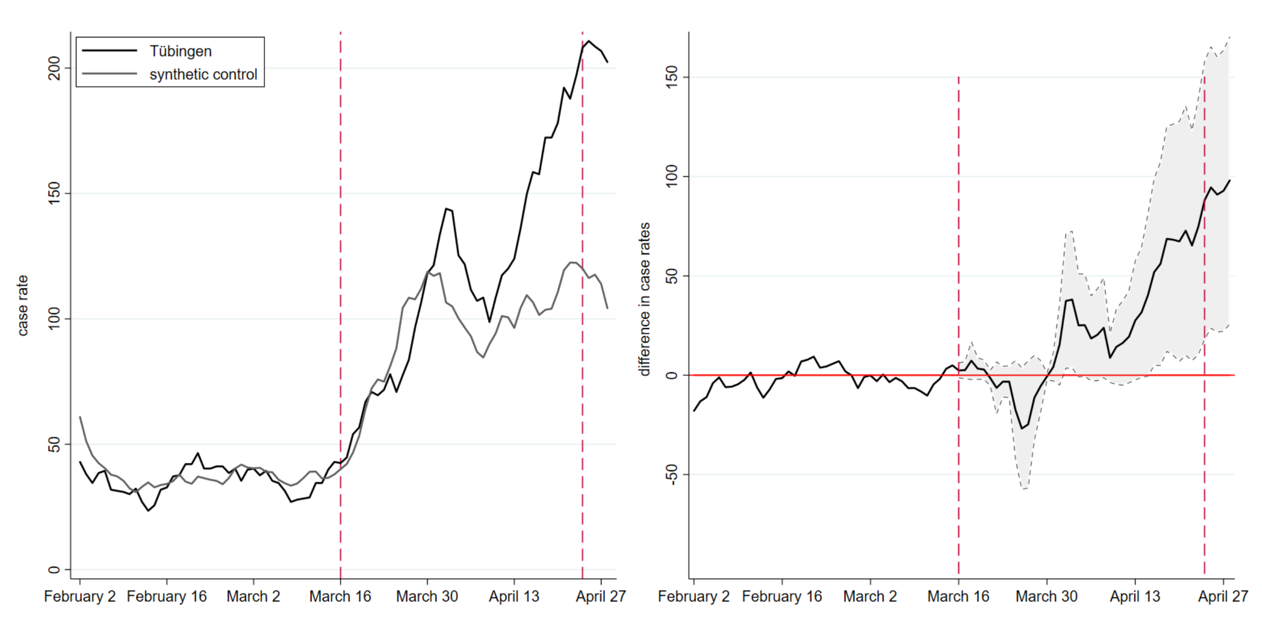

Given the visually similarly good fit in figure A8.13 and figure 1 raises the question why we opted for figure 1 as our baseline figure. There is a quantitative and a conceptional answer.

The pre-treatment RMSPE for figure 1 is lower than for figure A8.13. Table A4.4 gives a value of 9.9 while table A8.13 gives a value of 19.3. More from a conceptional perspective, figure 1 matches the synthetic control group based on weekly observations of the case rate starting in February 2021 while the match in Figure

Table A8.12. Control counties and weights for figure A8.13

Name	Weight
SK Heidelberg	0.45
LK Eichstätt	0.35
SK Trier	0.06
SK Leipzig	0.06
LK Nordfriesland	0.04
LK Emsland	0.04

A8.13 is based on daily observations of the case rate starting in March 2021, keeping the other predictor variables the same. The daily inclusion of the case rate over two weeks leads to a higher weighting of the case rate by the SCM algorithm that comes at the expense of a lower weighting for the other predictor variables. When choosing the predictor variables, we felt that considering the case rate over a longer time period of six weeks in our baseline specification is more appropriate than considering the case rate for only two weeks as we did for figure A8.13 because the treatment period was also nearly six weeks long.

We believe that same trends in the case rate in the treatment and control group before the treatment are very important for a short term evaluation of the treatment effect. For a long term evaluation of the treatment effect (we are looking at a post treatment period of more than one month) other factors such as the share of highly educated persons in the regional population become more relevant. Given the quantitative RMSPE finding and this more general consideration, we chose figure 1 instead of figure A8.13 as our main specification.

Table A8.13. Balancing properties of predictor set 1 for figure A8.13

	Treated	Synthetic
Seven-day case rate per 100k (Mar 1)	39.87	43.70
Seven-day case rate per 100k (Mar 2)	40.31	43.21
Seven-day case rate per 100k (Mar 3)	37.65	42.31
Seven-day case rate per 100k (Mar 4)	39.42	39.44
Seven-day case rate per 100k (Mar 5)	35.44	39.05
Seven-day case rate per 100k (Mar 6)	34.55	35.45
Seven-day case rate per 100k (Mar 7)	31.45	33.77
Seven-day case rate per 100k (Mar 8)	27.02	33.02
Seven-day case rate per 100k (Mar 9)	27.91	33.58
Seven-day case rate per 100k (Mar 10)	28.35	36.40
Seven-day case rate per 100k (Mar 11)	28.79	39.95
Seven-day case rate per 100k (Mar 12)	34.55	40.45
Seven-day case rate per 100k (Mar 13)	34.55	39.06
Seven-day case rate per 100k (Mar 14)	39.87	39.65
Seven-day case rate per 100k (Mar 15)	42.97	40.88
Cumulative cases over previous 7 days (Mar 9)	63.00	69.45
Cumulative cases over previous 14 days (Mar 15)	158.00	152.29
Average mobility (March 9 - March 15)	0.00	-0.16
Average temperature (March 9 - March 15)	4.21	6.10
Population density	434.86	880.89
Share of females in population	51.26	50.70
Average age of female population	41.67	41.98
Average age of male population	40.03	39.95
Old-age dependency ratio	24.58	25.21
Young-age dependency ratio	20.20	19.37
Medical doctors per population	15.64	18.98
Pharmacies per population	23.48	27.57
Categorial variable for population density of NUTS3 region	2.00	1.94
Share of highly educated persons in regional population	26.47	27.77
Stringency Index	2.97	2.97
Neighborhood (50km) seven-day case rate per 100k (Mar 1)	58.36	57.70
Neighborhood (50km) seven-day case rate per 100k (Mar 2)	58.16	57.84
Neighborhood (50km) seven-day case rate per 100k (Mar 3)	59.28	58.54
Neighborhood (50km) seven-day case rate per 100k (Mar 4)	58.90	58.14
Neighborhood (50km) seven-day case rate per 100k (Mar 5)	57.71	57.90
Neighborhood (50km) seven-day case rate per 100k (Mar 6)	59.75	57.13
Neighborhood (50km) seven-day case rate per 100k (Mar 7)	56.78	56.14
Neighborhood (50km) seven-day case rate per 100k (Mar 8)	56.70	56.68
Neighborhood (50km) seven-day case rate per 100k (Mar 9)	57.05	57.71
Neighborhood (50km) seven-day case rate per 100k (Mar 10)	61.97	58.48
Neighborhood (50km) seven-day case rate per 100k (Mar 11)	62.63	62.78
Neighborhood (50km) seven-day case rate per 100k (Mar 12)	65.31	66.46
Neighborhood (50km) seven-day case rate per 100k (Mar 13)	68.59	69.95
Neighborhood (50km) seven-day case rate per 100k (Mar 14)	69.39	74.28
Neighborhood (50km) seven-day case rate per 100k (Mar 15)	70.73	77.31
RMSPE (pre-treatment)	19.30	

Note: Dates in parentheses indicate when the respective variable was measured.

A8.2 Full donor pool and predictor set 2

We now consider a further alternative to the baseline predictor set employed for figure 1. Our predictor set 2 is shown in table A8.15 and puts less weight on case rates in the matching period. Not surprisingly, we get a different set of comparison counties in table A8.14 and a modified predictor balance (table A8.15). The result is in figure A8.14.

Table A8.14. Control counties and their weights for figure A8.14

Name	Weight
LK Eichstätt	0.45
SK Heidelberg	0.31
SK Freiburg i.Breisgau	0.17
LK Tuttlingen	0.07
SK Berlin	0.003

Table A8.15. Balancing properties of predictor set 2 for figure A8.14

	Treated	Synthetic
Seven-day case rate per 100k (Mar 1)	39.87	40.84
Seven-day case rate per 100k (Mar 8)	27.91	35.40
Seven-day case rate per 100k (Mar 15)	42.97	39.98
Cumulative cases over previous 7 days (Mar 9)	63.00	65.79
Cumulative cases over previous 14 days (Mar 15)	158.00	138.40
Average mobility (March 9 - March 15)	0.00	-0.13
Average temperature (March 9 - March 15)	4.21	5.64
Population density	434.86	783.67
Share of females in population	51.26	50.57
Average age of female population	41.67	41.71
Average age of male population	40.03	39.83
Old-age dependency ratio	24.58	24.67
Young-age dependency ratio	20.20	20.25
Medical doctors per population	15.64	18.22
Pharmacies per population	23.48	25.90
Categorial variable for population density of NUTS3 region	2.00	1.97
Share of highly educated persons in regional population	26.47	26.09
Stringency index	2.97	2.99
Neighborhood (50km) seven-day case rate per 100k (Mar 1)	58.36	47.05
Neighborhood (50km) seven-day case rate per 100k (Mar 8)	57.05	52.26
Neighborhood (50km) seven-day case rate per 100k (Mar 15)	70.73	72.11
RMSPE (pre-treatment)	20.00	

Note: Dates in parentheses indicate when the respective variable was measured.

As in figure 1, the left panel in figure A8.14 shows case rates in Tübingen (solid) and in the synthetic control county (dashed). The right panel shows their difference. As for predictor set 1, we get similar overall results. Again, our baseline specification is confirmed.

Figure A8.14. Seven-day case rates for predictor set 2

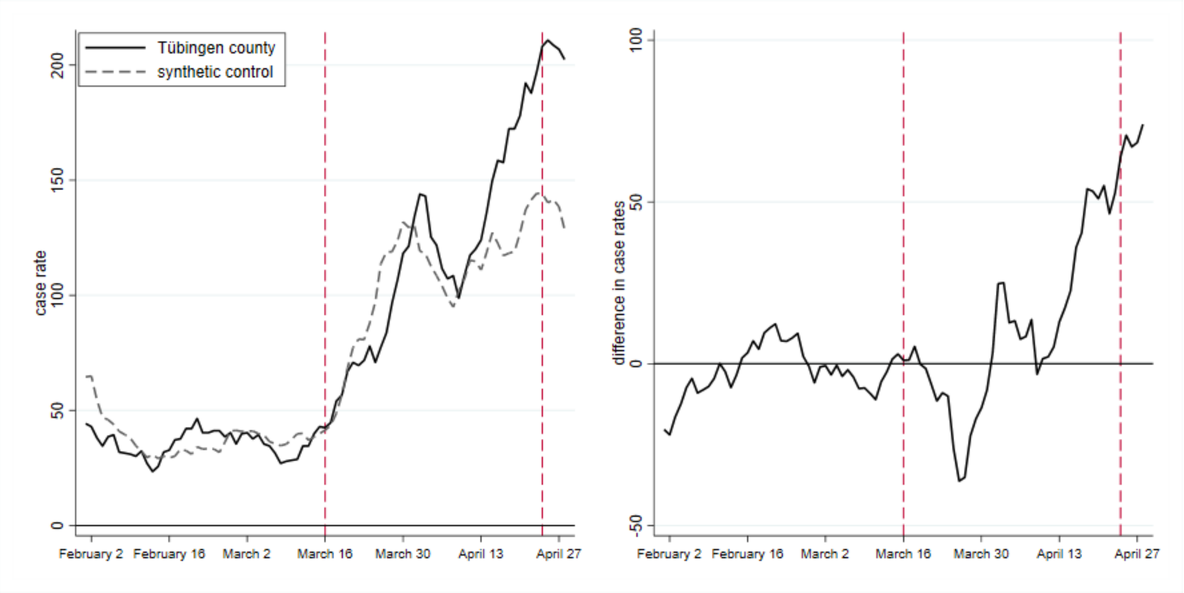

A8.3 The effect of spatial controls

One potential alternative explanation of why we see an increase in reported infection rates in Tübingen but only to a much smaller extent in the synthetic control could be that the pandemic development in neighboring counties affects the development in Tübingen. If, e.g., Tübingen before the beginning of OuS was surrounded by counties with high case rates, it is possible that these infections may spill over to Tübingen county. One key mechanism through which this may happen in the absence of OuS is through commuters. If that was the case, we would potentially observe an increase in case rates in Tübingen that is not due to OuS, but rather due to Tübingen's proximity to regions with high infection rates.

To check for this potential explanation, our baseline regression matches Tübingen with potential control regions that exhibit similar case rates in their respective neighboring counties. To this end, we construct a new, additional set of predictors, to which we refer as spatial predictors. For every week in our pre-treatment period and for every county in Germany, we calculate the average infection rates (reported infections over 7 days per 100,000 inhabitants) across neighboring counties. We define neighboring counties as all counties whose centroids are not more than 50 km away from the focal county's centroid. Using other definitions for example adjacent neighbors or a 90 km centroid definition did not change our results.

Our robustness check in figure 2 shows, again, that the main result does not change. We therefore conclude that the increases in cases in Tübingen relative to its synthetic twin is not driven by infection rates in neighbouring counties creating a surge in cases in Tübingen.

A9 Further robustness checks

A9.1 Robustness check for figure A8.13

Figure A9.15 demonstrates that findings in figure A8.13 do not strongly depend on the exact composition of the synthetic control group. When counties employed for figure A8.13, listed in table A8.12, are excluded one-by-one, hardly any change is visible. In fact, changes induced by the exclusion of most control counties are so small that the lines of the different synthetic control groups overlap to a great extent.

Figure A9.15. Leave one out for figure A8.13

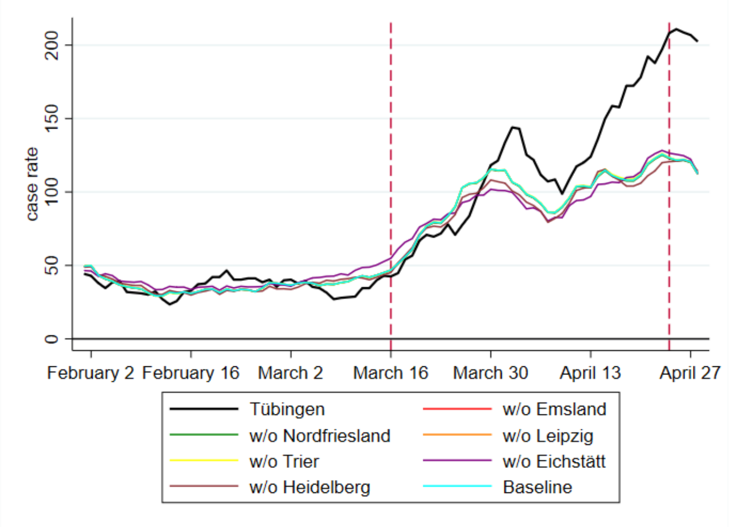

A9.2 Difference-in-difference estimation

This section reports the results of a series of additional robustness tests for our main SCM results using difference-in-difference (DiD) estimation.

- Estimation setup

DiD estimation is one of the most frequently applied tools for impact evaluation studies and, hence, the method has also been extensively used to identify policy effects during the COVID-19 crisis. In several cases, DiD and SCM estimation have been combined in a multiple method approach to deliver robust effect estimates that serve as input for the public policy debate (examples include [12], [21], [33], [10]). One advantage of the DiD estimator, which makes its application attractive for the analysis at hand, is that it allows us to explicitly control for heterogeneous time trends in SARS-CoV-2 infection dynamics at the local level during the pre- and also during the treatment period that are not attributable to the treatment.

As outlined in [33], the DiD approach – similar to SCM – estimates treatment effects by contrasting changes in outcomes between a pre-treatment and treatment period for treated and non-treated (control) units. DiD estimation can be regarded as being more flexible than SCM estimation in terms of controlling for heterogeneous deterministic time trends and in testing for dynamic treatment effects over cross-sections and across time. At the same time, the approach has its limits when only one (as in our case) or very few treated regions are available as heteroscedastic errors might occur [20].

Moreover, the consistency of treatment effects estimated in a DiD framework chiefly depends on the validity of common pre-treatment trends for treated and non-treated units. This is different from SCM, which does not need to impose a common pre-trend assumption as the presence of common trends between treated and control regions is in itself a favorable factor for finding an appropriate counterfactual trajectory ([5], [4], [2], [32]).

- Implementation

In order to utilize the strength of DiD estimation for robustness tests while controlling for its potential weaknesses, we estimate alternative DiD specifications. These differ by the extent to which they allow for heterogeneous time trends and how the control group of non-treated regions is composed. In both cases, modelling choices may affect the ability of DiD estimation to fulfill the common pre-trend assumption. We will assess the latter by an inspection of the size and statistical significance of placebo treatment effects prior to treatment start.

Our baseline specification (denoted DiD1) has the following form:

$$case\_rate_{i,t} = \mu_i + \sum_{m=t_0}^{T} \delta_m \times reopen_{i,t}^m + \tau_1 \times trend_t + \tau_2 \times trend_t^2 + \tau_3 \times trend_t^3 + e_{i,t}. \quad \text{(A9.15)}$$

In A9.15, $case\_rate_{i,t}$ is the model's outcome variable, i.e., the SARS-CoV-2 7-day case rate in district i at day t and μ_i are district-level fixed effects as a "catch-all" term for outcome differences associated with time-fixed factors at the local level, which may confound with outcome differences across district. In the SCM estimation, we have explicitly accounted for individual factors at the local district level such as the demographic composition of a district, its population density etc. Following the approach proposed in [10], we also include a set of linear and non-linear (quadratic and cubic) time trends in the baseline specification to capture the temporal dynamics of SARS-CoV-2 infection waves in Germany during our sample period. In the baseline specification, we assume that the estimated temporal dynamics is common to all districts.

Most notably, we identify dynamic treatment effects by the inclusion of a set of binary dummies $\sum reopen_{i,t}^m$. Thereby, the m-th dummy takes a value of one for Tübingen on day $m = t_0, \dots, T$, where t_0 marks the start of the re-opening experiment (i.e., the relaxation of lockdown measures) in t_0 and T is the last sample period. Hence, each individual coefficient δ_m measures the relative treatment effect for Tübingen vis-à-vis non-treated districts at day m of the treatment period. Plotting the individual coefficients together with a measure for their statistical significance allows us to identify the dynamic nature of treatment effects over time. By extending the set of dummies to cover days $p = t_0 - n, \dots, t_0 - 1$ additionally allows us to check for placebo treatment effects between $t_0 - n$ and the last day before treatment start. If the common pre-trend assumption of our DiD specification holds, we should not see significant coefficients for the p-th placebo dummy (as default we set $n = 10$, i.e., we test for early anticipation effects up to 10 days prior to treatment start).

As outlined above, one limitation of the baseline DiD specification is the imbalance between our single treatment unit (i.e., the district of Tübingen) and a relatively large control group comprising all other German districts (395 in total excluding direct neighboring districts). This imbalance between the size of the treatment and control group may result in imprecise treatment effect estimates and, importantly, lead to a violation of the common trend if the districts in the control group follow very different infection trends prior to the start of the re-opening experiment in Tübingen. We account for this potential bias by applying conditional DiD estimation in a two-step manner as proposed, e.g., in [33]. In a first step, we thereby estimate propensity score values for each district by running a cross-sectional Probit regression that models the likelihood of a control district to be similar to Tübingen at the onset of the reopening experiment, i.e., the last pre-treatment observation.

We include the following district-level characteristics in the Probit regression: 1) lagged levels of the case rate and its spatial lag term (one week), 2) lagged changes in the case rate and its spatial lag term (one and two weeks), 3) lagged mobility levels (one week), 4) lagged changes in mobility (one and two weeks), together with 5) regional characteristics in analogy to those used for control groups selection in the SCM approach (i.e., average temperature, demographic characteristics, stringency index). We use the obtained propensity score values to downscale the control group of non-treated districts to those with only positive propensity score values. In line with SCM estimation, the logic of this scaling exercise is to make the control group more similar to Tübingen with respect to epidemiological trends before treatment start and other regional characteristics that may influence the SARS-CoV-2 development throughout the treatment period. In result, this reduces the control group from 395 to 55 districts (see Table A9.16 for non-treated districts included in the control group). We then estimate A9.15 for this subsample (denoted DiD2).

Table A9.16. Subsample of German districts selected by first-step Probit estimation

ID	Name	ID	Name
1061	LK Steinburg	9184	LK München
3101	SK Braunschweig	9188	LK Starnberg
3155	LK Northeim	9377	LK Tirschenreuth
3159	LK Göttingen	9464	SK Hof
3254	LK Hildesheim	9478	LK Lichtenfels
3255	LK Holzminden	9479	LK Wunsiedel i.Fichtelgebirge
3404	SK Osnabrück	9573	LK Fürth
3451	LK Ammerland	12051	SK Brandenburg a.d.Havel
3455	LK Friesland	12054	SK Potsdam
6411	SK Darmstadt	12062	LK Elbe-Elster
6414	SK Wiesbaden	13003	SK Rostock
6434	LK Hochtaunuskreis	14521	LK Erzgebirgskreis
6439	LK Rheingau-Taunus-Kreis	14523	LK Vogtlandkreis
7211	SK Trier	14612	SK Dresden
7231	LK Bernkastel-Wittlich	14627	LK Meißen
7232	LK Bitburg-Prüm	14628	LK
7235	LK Trier-Saarburg	15003	SK Magdeburg
7336	LK Kusel	15085	LK Harz
7339	LK Mainz-Bingen	15086	LK Jerichower Land
8211	SK Baden-Baden	16051	SK Erfurt
8221	SK Heidelberg	16053	SK Jena
8326	LK Schwarzwald-Baar-Kreis	16054	SK Suhl
8327	LK Tuttlingen	16055	SK Weimar
8335	LK Konstanz	16062	LK Nordhausen
8416	LK Tübingen	16066	LK Schmalkalden-Meiningen
8435	LK Bodenseekreis	16071	LK Weimarer Land
9171	LK Altötting	16076	LK Greiz
9175	LK Ebersberg		

Notes: LK = Landkreis; SK = Stadtkreis. Districts (Land- and Stadtkreise) correspond to NUTS Level 3 regions in the classification of territorial units of the European Union.

Another limitation of the baseline DiD specification is that the temporal dynamics of SARS-CoV-2 infection waves have not been symmetric across the different German regions during our sample period. To avoid biases stemming from these different infection trends, we further extend the DiD model as shown in A9.15 by interacting the included time trends with dummies for the 16 German federal states. As general public health regulations have differed across German federal states but were homogeneous within federal states (except for notable exceptions as the re-opening exercise in Tübingen), we argue that this is an important channel to account for spatial heterogeneity in SARS-CoV-2 infection trends during our sample period. We thus extend A9.15 as

$$case\_rate_{i,t} = ... + \sum_{j=1}^{16} \tau_{1,j} \times (trend_t \times state_{j(i)}) + \sum_{j=1}^{16} \tau_{2,j} \times (trend_t^2 \times state_{j(i)}) + \sum_{j=1}^{16} \tau_{3,j} \times (trend_t^3 \times state_{j(i)}) + ... + e_{i,t}, \quad \text{(A9.16)}$$

where $state_{j(i)}$ is a dummy for the j-th federal state that takes a value of one if district i is located in state j. While the obtained interaction terms are hence able to capture different infection dynamics across federal states, as noted in [10], a potential downside of this approach is that the estimated treatment effects may be too low if state level trends confound with treatment effects. We thus regard the latter estimates

as lower bounds for treatment effects of Tübingen vis-à-vis the average development in the federal state of Baden-Württemberg in which Tübingen is located in. We combine conditional DiD estimation with state-specific time trends as third DiD specification applied here (denoted DiD3).

- Results

Figure A9.16 shows the estimated daily treatment effects for the reopening experiment in Tübingen according to our three specifications DiD1, DiD2 and DiD3. In the figure, the different symbols show the point estimates for δ_m in each specification. As for the visual inspection of the SCM results, the first vertical line (seen from the left side) denotes the start of the re-opening experiment on March 16, 2021. The second vertical line marks the termination of the experiment after April 24, 2021. With regard to effect size and its temporal evolution, the DiD results lend further support to our main SCM findings. In all three cases, we find that effects gradually build up over time with temporary drop in effects during the Easter break.

Figure A9.16. Robustness checks for treatment effects in Tübingen using DiD estimation

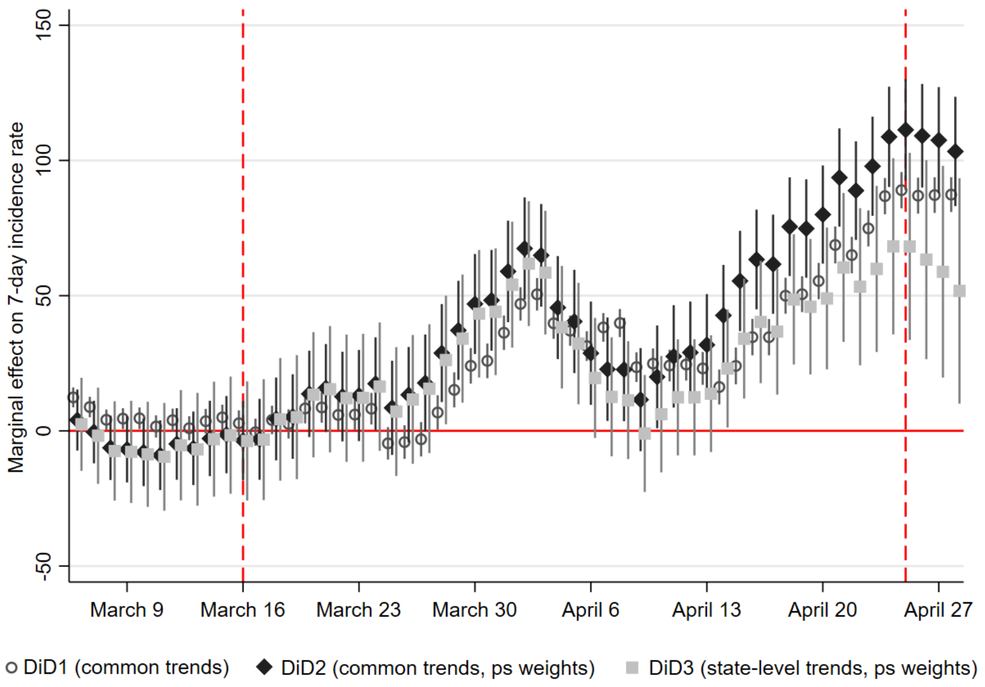

Notes: Symbols show point estimates and spikes indicate 95% confidence intervals for robust standard errors clustered at the district level. While DiD1 uses the full sample of German districts to estimate daily treatment effects, DiD2 and DiD3 are estimated for a subsample of control districts listed in Table A9.16 that were selected using propensity scores. DiD3 additionally includes state specific time trends.

Both the baseline DiD specification and the propensity score-based subsample report marginal effects in terms of cases rate differences between Tübingen and the average case rate in the group of non-treated regions of about 100 additional cases per 100,000 inhabitants (evaluated as of April 24, 2021). This is in line with our main SCM estimates reported in Figure 1. While we get some evidence against common pre-trends between Tübingen and the control group in the DiD1 specification, there is no sign of early anticipation effects when we look at the estimated daily treatment effects before treatment start in the DiD2 and DiD3 specifications.

The latter specification incorporating state-specific time trends also reports more conservative treatment effect of max. 50 additional cases per 100,000 inhabitants. One has to note, though, that the reference case are now non-treated districts in the same federal state as Tübingen is located in (Baden-Württemberg). If we compare this effect size with the corresponding SCM results for the subsample of districts located in

Baden-Württemberg in Figure 2, both estimation methods report almost identical results. Taken together, we argue that the DiD-based robustness tests lend further support to our main SCM findings.

A10 Does Tübingen city differ from Tübingen county?

The final check of our findings consists in treating the *city* of Tübingen as an independent unit. The county of Tübingen is then also excluded from the donor pool, as are neighboring regions of the county of Tübingen (as in all other donor pools). Figure A10.17 provides some geographical background.

Figure A10.17. The location of Tübingen county within Germany (left) and its communities (right, figures generated employing [22])

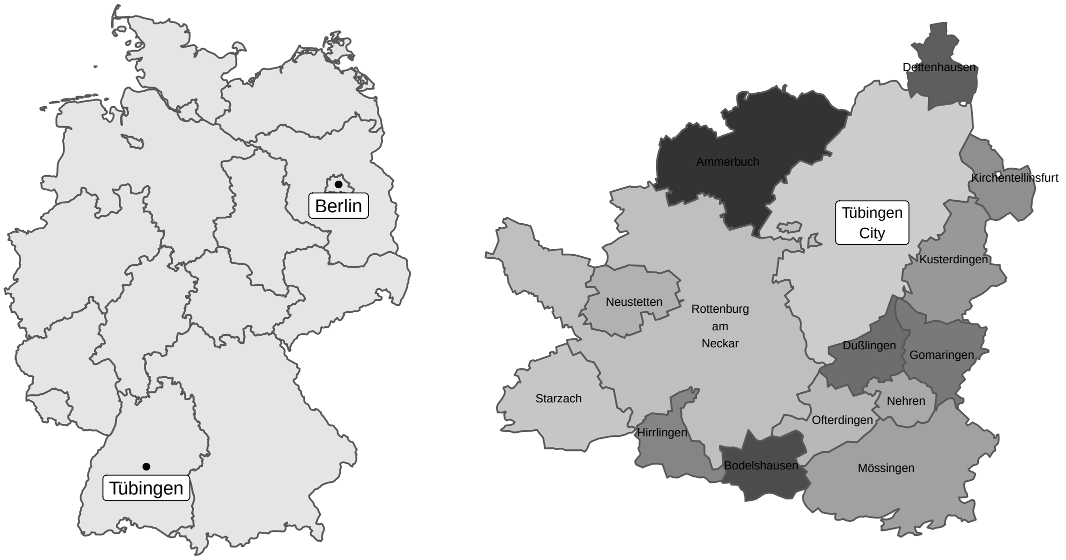

The effect of OuS on Tübingen city can be understood only with an adjusted predictor set. Data used in our predictor sets used so far are not all available for Tübingen city. We therefore collected information on Tübingen city and grouped all available variables in our new predictor set 'community' shown in table A10.18. Variables found for Tübingen city were then also used for all other counties in Germany.

A10.1 The effect of the predictor set community for Tübingen county

Imagine we find some result on Tübingen city that differs from our baseline finding. Given that we changed both the region of analysis and the predictor set, we would not know whether the differential effect is due to our focus on Tübingen city. It could be due to the new predictor set 'community'.

To rule this out, we now replicate our baseline analysis with the predictor set 'community' instead of the predictor set 'baseline'. The resulting synthetic control group is described in table A10.17.

Table A10.17. Weights for figure A10.18 (left)

name	weight
LK Eichstätt	0.38
LK Lüneburg	0.20
SK Oldenburg	0.12
SK Brandenburg a.d.Havel	0.09
SK Rostock	0.07
SK Heidelberg	0.05
SK Neustadt a.d.Weinstraße	0.02
SK Frankenthal	0.02
SK Erlangen	0.02
LK Ebersberg	0.01
SK Osnabrück	0.01
LK Steinburg	0.01
LK Kusel	0.01

Table A10.18. Balancing properties of predictor set community for figure A10.18

	Treated	Synthetic
Seven-day case rate per 100k (Feb 9)	31.01	31.25
Seven-day case rate per 100k (Feb 15)	31.89	31.98
Seven-day case rate per 100k (Feb 22)	40.31	40.21
Seven-day case rate per 100k (Mar 1)	39.87	39.91
Seven-day case rate per 100k (Mar 8)	27.02	27.53
Seven-day case rate per 100k (Mar 15)	42.97	42.77
Cumulative cases over previous 7 days (Mar 15)	97.00	59.38
Population density	434.86	516.46
Share of females in population	51.26	50.50
Young-age dependency ratio	20.20	20.94
Old-age dependency ratio	24.58	29.79
Incommuting	64.86	64.62
Outcommuting	67.73	63.90
Accessibility	13.00	15.56
Pharmacies per population	23.48	24.25
Transport	99.00	89.71
Stringency index	2.91	2.89
Neighborhood (50km) seven-day case rate per 100k (Feb 8)	61.62	61.52
Neighborhood (50km) seven-day case rate per 100k (Feb 15)	51.14	52.02
Neighborhood (50km) seven-day case rate per 100k (Feb 22)	44.51	51.10
Neighborhood (50km) seven-day case rate per 100k (Mar 1)	58.36	54.75
Neighborhood (50km) seven-day case rate per 100k (Mar 8)	56.70	54.28
Neighborhood (50km) seven-day case rate per 100k (Mar 15)	70.73	70.75
RMSPE (pre-treatment)	3.19	

Note: Dates in parentheses indicate when the respective variable was measured.

The left part of figure A10.18 shows us the effect of changing the predictor set from 'baseline' to 'community'. The right panel replicates our baseline figure 1. As we see, the effect of OuS under the predictor set 'community' appears somewhat larger than in the baseline scenario. Practically speaking, however, the findings are the same. Hence, any change we might find in what follows for the city of Tübingen as compared to Tübingen county cannot be due to the use of the new predictor set 'community'.

Figure A10.18. Tübingen county with predictor set 'community' (left) and baseline (right)

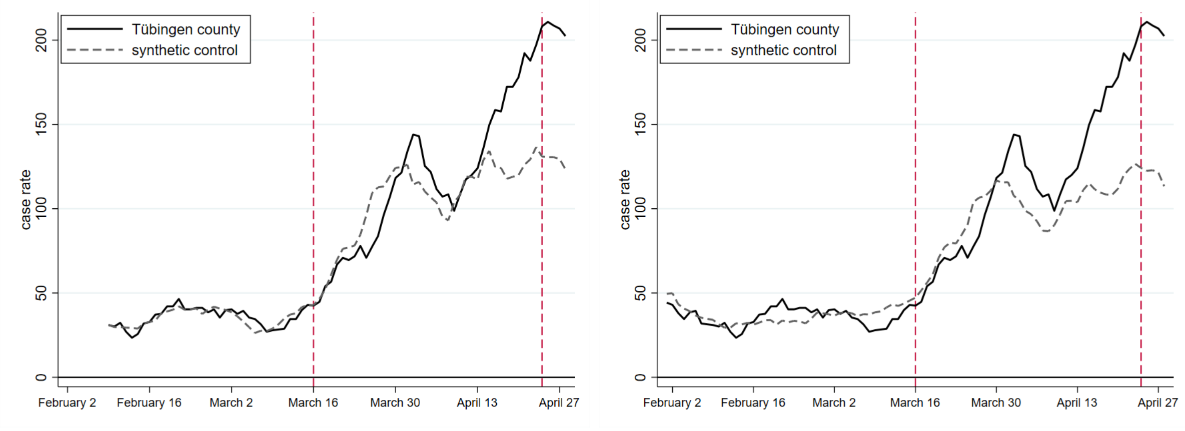

A10.2 Studying growth rates: Normalization

Finding control counties for Tübingen city with seven-day case rates as the pandemic measure turned out to be very difficult. This is due to the fact that Tübingen city had very low case rates (as shown earlier in figure A2.2). The pre-treatment fit and the predictor balance were very bad in an initial SCM analysis. This might also be due to the fact that SCM allows for positive weights only. We therefore normalized seven-day case rates such that they equal zero on March 16 for all counties (and for Tübingen city, as also shown earlier in figure A2.3).

- Tübingen county

We now re-perform our baseline SCM with these normalized case rates. Figures then display growth factors of cases rates (or growth rates if normalized by 100). Figure A10.19 plots time on the horizontal and growth factors on the vertical axis. It shows that case rates in Tübingen county increased by a factor of 4 as compared to an increase in case rates in the synthetic control county by a factor of 2. When we restrict the donor pool to Baden-Württemberg in the right panel, the same ratios can be found. Hence, working with growth rates of case rates as opposed to case rates in our baseline analysis leads to the same overall conclusion that we stress in the main text. This allows us to finally study the city of Tübingen.

Figure A10.19. Growth factors for Tübingen county and synthetic control group. Donor pool Germany (left) and Baden-Württemberg (right)

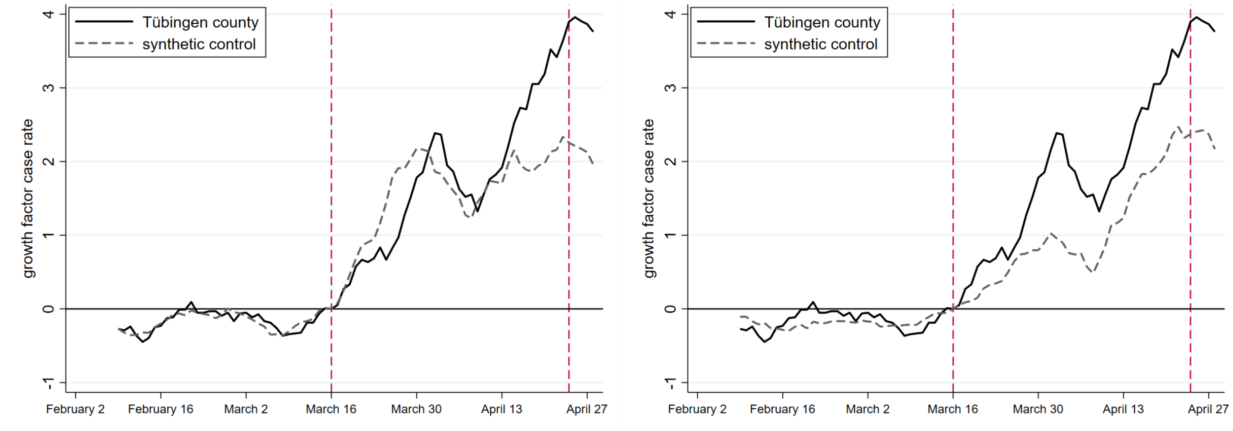

Table A10.19. Weights for figure A10.19 left and right

name	weight
LK Eichstätt	0.23
SK Osnabrück	0.16
LK Dachau	0.16
LK Lüneburg	0.10
SK Brandenburg a.d.Havel	0.08
SK Oldenburg	0.08
LK Konstanz	0.07
LK Ebersberg	0.06
SK Landshut	0.02
LK Plön	0.02
SK Bottrop	0.01

name	weight
LK Enzkreis	0.41
SK Ulm	0.17
LK Konstanz	0.13
SK Heidelberg	0.13
LK Rhein-Neckar-Kreis	0.11
LK Heilbronn	0.05
LK Rottweil	0.001

- Tübingen City

Having shown that replacing the baseline predictor set by a predictor set community and that working with normalized case rates instead of case rates yields the same findings for Tübingen county, we can now study Tübingen city by employing the predictor set community and normalized case rates. The results are in figure A10.20, the predictor balance table follows thereafter, as does the table with control counties.

This figure clearly shows that the growth in the case rate in Tübingen city is much larger than in the control county. The right panel shows that the increase is higher by a factor of around 3.

We conclude from this that there are effects in Tübingen city as well. The conclusion that the rise in cases in Tübingen county is solely due to an increase in cases outside of Tübingen city would not be correct.

Figure A10.20. Growth factors for Tübingen city and synthetic control group

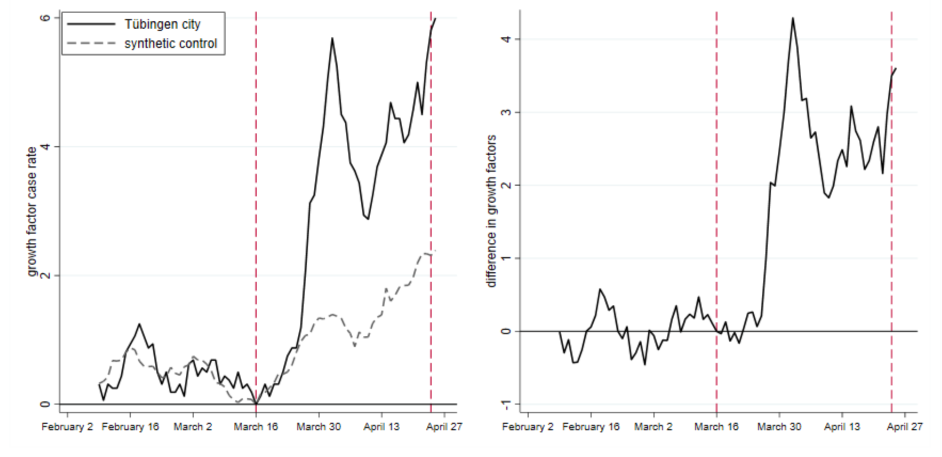

Table A10.20. Balancing properties of predictor set community for figure A10.20

	Treated	Synthetic
Seven-day case rate per 100k (Feb 8)	0.31	0.32
Seven-day case rate per 100k (Feb 15)	0.81	0.81
Seven-day case rate per 100k (Feb 22)	0.50	0.50
Seven-day case rate per 100k (Mar 1)	0.63	0.61
Seven-day case rate per 100k (Mar 8)	0.31	0.32
Seven-day case rate per 100k (Mar 15)	0.19	0.07
Cumulative cases over previous 7 days (March 15)	19.00	53.64
Population density	827.00	733.88
Share of females in population	52.50	51.00
Young-age dependency ratio	16.90	18.83
Old-age dependency ratio	20.40	35.53
Incommuting	62.10	61.61
Outcommuting	43.40	51.13
Accessibility	0.00	21.79
Pharmacies per population	80.00	34.65
transport	100.00	95.45
Stringency index	2.91	2.80
Neighborhood (50km) seven-day case rate per 100k (Feb 9)	61.62	68.62
Neighborhood (50km) seven-day case rate per 100k (Feb 15)	51.14	56.46
Neighborhood (50km) seven-day case rate per 100k (Feb 22)	44.51	58.10
Neighborhood (50km) seven-day case rate per 100k (Mar 1)	58.36	58.73
Neighborhood (50km) seven-day case rate per 100k (Mar 8)	56.70	59.86
Neighborhood (50km) seven-day case rate per 100k (Mar 15)	70.73	73.99
RMSPE (pre-treatment)	0.27	

Note: Dates in parentheses indicate when the respective variable was measured.

Table A10.21. Weights for figure A10.20

Name	Weight
SK Baden-Baden	0.30
SK Kiel	0.22
LK Merzig-Wadern	0.10
LK Rottweil	0.01
LK Cochem-Zell	0.08
LK Northeim	0.06
LK Goslar	0.05
SK Flensburg	0.04
SK Straubing	0.03
SK Weiden i.d.OPf.	0.03

A11 The future of Opening under Safety

At the moment these lines are written, the world experiences the omicron-wave of the pandemic. Various countries returned to policies that require rapid testing to allow social contacts e.g. in restaurants. Given the renewed interest in testing, what do we learn from the Tübingen experience? How do our findings compare to more successful OuS projects as reported in [17]?

First, the link between testing centers and health authorities must be strengthened. Positive rapid testing must be PCR confirmed and reported back to testing centers. Data from testing centers would then be much more informative. Second, infection data at the community level should be made public in a systematic way.

Third, data on individuals that participate in OuS projects should be collected. Our evaluation is based on aggregate county and community data. It would be much more informative if we knew whether individuals taking part in OuS are actually infected around 5-7 days after the event they took part in. Such a 'terminal test' joint with the initial rapid test would allow to draw conclusions about OuS much more convincingly. If the participation of individuals in individual events was also registered (for some examples, see [17]), one could also draw conclusions about individual components of OuS (is going to the cinema more risky than going shopping?).

Comparing OuS in Augustusburg with OuS in Tübingen, some conjectures can be made which, at this point, require more data to be confirmed. This discussion also relates to the 'catching up' hypothesis laid out above. Why could 'catching up' ever take place? Only when testing is not perfect. It is clear that any testing procedure produces false negatives. It is also clear that not all infected and infectious individuals can be detected by rapid testing [28]. Hence, testing centers, their management and their personnel is crucial in keeping the real-world (as opposed to laboratory) number of false negatives low. We have no data whatsoever to claim that testing centers were at the basis of the rise of infections in this OuS project. Other hypotheses should be taken into account as well in case future OuS projects will take place and will be evaluated.

References

[1] Infas 360. *Erfasste Verordnungen und Maßnahmen in Kategorien und Unterkategorien.* 2021. URL: https://www.corona-datenplattform.de/.

[2] Alberto Abadie. "Using Synthetic Controls: Feasibility, Data Requirements, and Methodological Aspects". In: *Journal of Economic Literature* (2020). URL: https://www.aeaweb.org/content/file?id=12409.

[3] Alberto Abadie, Alexis Diamond, and Jens Hainmueller. *SYNTH: Stata module to implement Synthetic Control Methods for Comparative Case Studies.* Statistical Software Components, Boston College Department of Economics. Oct. 2011. URL: https://ideas.repec.org/c/boc/bocode/s457334.html.

[4] Alberto Abadie, Alexis Diamond, and Jens Hainmueller. "Synthetic Control Methods for Comparative Case Studies: Estimating the Effect of California's Tobacco Control Program". In: *Journal of the American Statistical Association* 105.490 (2010), pp. 493–505. DOI: 10.1198/jasa.2009.ap08746. eprint: https://doi.org/10.1198/jasa.2009.ap08746. URL: https://doi.org/10.1198/jasa.2009.ap08746.

[5] Alberto Abadie and Javier Gardeazabal. "The Economic Costs of Conflict: A Case Study of the Basque Country". In: *American Economic Review* 93.1 (2003), pp. 113–132. DOI: 10.1257/000282803321455188. URL: https://www.aeaweb.org/articles?id=10.1257/000282803321455188.

[6] Douglas G Altman and J Martin Bland. "How to obtain the confidence interval from a P value". In: *BMJ* 343 (2011). DOI: 10.1136/bmj.d2090. eprint: https://www.bmj.com/content/343/bmj.d2090.full.pdf. URL: https://www.bmj.com/content/343/bmj.d2090.

[7] Rachel E. Baker et al. "Assessing the influence of climate on wintertime SARS-CoV-2 outbreaks". In: *Nature Communications* 12 (2021), p. 846. ISSN: 2041-1723. DOI: https://doi.org/10.1038/s41467-021-20991-1. URL: https://doi.org/10.1038/s41467-021-20991-1.

[8] Benjamin Born, Alexander M. Dietrich, and Gernot J. Müller. "The lockdown effect: A counterfactual for Sweden". In: *PLOS ONE* 16.4 (Apr. 2021), pp. 1–13. DOI: 10.1371/journal.pone.0249732. URL: https://doi.org/10.1371/journal.pone.0249732.

[9] Benjamin Born et al. "The Costs of Economic Nationalism: Evidence from the Brexit Experiment". In: *The Economic Journal* 129.623 (May 2019), pp. 2722–2744. DOI: 10.1093/ej/uez020. URL: https://doi.org/10.1093/ej/uez020.

[10] Philipp Breidenbach and Timo Mitze. "Large-scale sport events and COVID-19 infection effects: evidence from the German professional football 'experiment'". In: *The Econometrics Journal* 25.1 (July 2021), pp. 15–45. ISSN: 1368-4221. DOI: 10.1093/ectj/utab021. eprint: https://academic.oup.com/ectj/article-pdf/25/1/15/42182859/utab021.pdf. URL: https://doi.org/10.1093/ectj/utab021.

[11] Eduardo Cavallo et al. "Catastrophic Natural Disasters and Economic Growth". In: *The Review of Economics and Statistics* 95.5 (2013), pp. 1549–1561. DOI: {https://doi.org/10.1162/REST_a_00413}. URL: https://doi.org/10.1162/REST_a_00413.

[12] Sang-Wook Cho. "Quantifying the impact of nonpharmaceutical interventions during the COVID-19 outbreak: The case of Sweden". In: *The Econometrics Journal* 23.3 (Aug. 2020), pp. 323–344.

[13] Jonas Dehning et al. "Inferring COVID-19 spreading rates and potential change points for case number forecasts". In: *Science* 369.6500 (2020).

[14] Deutscher Bundestag and Deutscher Bundesrat. *Viertes Gesetz zum Schutz der BEvölkerung bei einer epidemischen Lage von nationaler Tragweite*. 2021. URL: https://www.gesetze-im-internet.de/ifsg/IfSG.pdf.

[15] Deutscher Wetterdienst (German Weather Service). *Climate Data Center (CDC), General Website:* https://www.dwd.de/EN/climate_environment/cdc/cdc.html. Accessed: April 12, 2021. 2021.

[16] Marc Diederichs et al. "Is large-scale rapid CoV-2 testing a substitute for lockdowns? The case of Tübingen". In: *medRxiv* (2021). DOI: 10.1101/2021.04.26.21256094. eprint: https://www.medrxiv.org/content/early/2021/04/26/2021.04.26.21256094.full.pdf. URL: https://www.medrxiv.org/content/early/2021/04/26/2021.04.26.21256094.

[17] Marc Diederichs et al. "Testing & Opening in Augustusburg - A Success Story?" In: *https://www.macro.economics.uni-mainz.de/klaus-waelde/ongoing-work-and-publications/* (2021). URL: https://www.macro.economics.uni-mainz.de/files/2021/05/Evaluation_Augustusburg-Zwischenbericht-final.pdf.

[18] J. R. Donsimoni et al. "Projecting the Spread of COVID19 for Germany". In: *German Economic Review* 21 (2020), pp. 181–216.

[19] Martin Eichenbaum, Sergio Rebelo, and Mathias Trabandt. *The Macroeconomics of Testing and Quarantining*. mimeo. 2021.

[20] Bruno Ferman and Cristine Pinto. "Inference in Differences-in-Differences with Few Treated Groups and Heteroskedasticity". In: *The Review of Economics and Statistics* 101.3 (July 2019), pp. 452–467. ISSN: 0034-6535. DOI: {10.1162/rest_a_00759}. eprint: https://direct.mit.edu/rest/article-pdf/101/3/452/1916793/rest_a_00759.pdf. URL: https://doi.org/10.1162/rest%5C_a%5C_00759.

[21] Andrew I Friedson et al. *Did California's Shelter-in-Place Order Work? Early Coronavirus-Related Public Health Effects*. Tech. rep. NBER Working Paper 26992, Apr. 2020.

[22] GADM. *Administrative Boundary Data 3.6, Germany*. 2021.

[23] Sebastian Galiani and Brian Quistorff. "The Synth_Runner Package: Utilities to Automate Synthetic Control Estimation Using Synth". In: *The Stata Journal* 17.4 (2017), pp. 834–849. DOI: 10.1177/1536867X1801700404. eprint: https://doi.org/10.1177/1536867X1801700404. URL: https://doi.org/10.1177/1536867X1801700404.

[24] Giorgia Guglielmi. *Rapid coronavirus tests: a guide for the perplexed*. Nature, news feature. 2021. DOI: doi.org/10.1038/d41586-021-00332-4.

[25] Thomas Hale et al. *Variation in government responses to COVID-19*. Tech. rep. Blavatnik School Working Paper WP-2020/032, 2020.

[26] H.W. Hethcote. "The Mathematics of Infectious Diseases". In: *SIAM Review* 42.4 (2000), pp. 559–653.

[27] INKAR. *Indikatoren und Karten zur Raum- und Stadtentwicklung: Bundesinstitut für Bau-, Stadt- und Raumforschung (Federal Institute for Research on Building, Urban Affairs and Spatial Development), General Website:* https://www.inkar.de/. Accessed: June 4, 2020. 2021.

[28] T. C. Jones et al. "Estimating infectiousness throughout SARS-CoV-2 infection course". In: *Science* (2021). DOI: 10.1126/science.abi5273. eprint: https://science.sciencemag.org/content/early/2021/05/24/science.abi5273.full.pdf.

[29] Monita Karmakar, Paula M. Lantz, and Renuka Tipirneni. "Association of Social and Demographic Factors With COVID-19 Incidence and Death Rates in the US". In: *JAMA Network Open* 4.1 (Jan. 2021), e2036462–e2036462. ISSN: 2574-3805. DOI: 10.1001/jamanetworkopen.2020.36462. eprint: https://jamanetwork.com/journals/jamanetworkopen/articlepdf/2775732/karmakar_2021_oi_201090_1611286825.95028.pdf. URL: https://doi.org/10.1001/jamanetworkopen.2020.36462.

[30] Stephen M. Kissler et al. "Reductions in commuting mobility correlate with geographic differences in SARS-CoV-2 prevalence in New York City". In: *Nature Communications* 11 (2021), p. 4674. ISSN: 2041-1723. DOI: https://doi.org/10.1038/s41467-020-18271-5. URL: https://doi.org/10.1038/s41467-020-18271-5.

[31] Kevin Kloiber et al. "Städte sind stärker von den Coronakrise betroffen Coronakrise betroffen". In: *ifo Schnelldienst* 5.April (2021). URL: https://www.ifo.de/node/62843.

[32] Noemi Kreif et al. "Examination of the Synthetic Control Method for Evaluating Health Policies with Multiple Treated Units". In: *Health economics* 25 (Oct. 2015). DOI: 10.1002/hec.3258.

[33] Timo Mitze et al. "Face masks considerably reduce COVID-19 cases in Germany". In: *Proceedings of the National Academy of Sciences* 117.51 (2020), pp. 32293–32301. ISSN: 0027-8424. DOI: https://doi.org/10.1073/pnas.2015954117. URL: https://www.pnas.org/content/117/51/32293.

[34] Boris Palmer and Lisa Federle. "Projekt Öffnen mit Sicherheit: Zweiter Zwischenbericht". In: *Stadt Tübingen: Der Oberbürgermeister* (2021). URL: www.tuebingen.de/Dateien/modellprojekt_zweiter_zwischenbericht_land.pdf.

[35] Robert Koch Institute. *Covid-19 Infektionen, General Website (NPGEO Corona Hub):* https://npgeo-corona-npgeo-de.hub.arcgis.com/. Accessed: February 8, 2021. 2021.

[36] Paul Romer. *Roadmap to responsibly reopen America.* Tech. rep. 2020. URL: https://roadmap.paulromer.net/paulromer-roadmap-report.pdf.

[37] Frank Schlosser et al. "COVID-19 lockdown induces disease-mitigating structural changes in mobility networks". In: *Proceedings of the National Academy of Sciences* 117.52 (2020), pp. 32883–32890. ISSN: 0027-8424. DOI: 10.1073/pnas.2012326117. eprint: https://www.pnas.org/content/117/52/32883.full.pdf. URL: https://www.pnas.org/content/117/52/32883.

[38] The University of Liverpool et al. *Liverpool Covid-19 Community Testing Pilot: Interim Evaluation Report.* Tech. rep. December. Liverpool: The University of Liverpool, 2020. URL: https://www.liverpool.ac.uk/media/livacuk/coronavirus/Liverpool,Community,Testing,Pilot,Interim,Evaluation.pdf.
